# Effect of Seasoning Addition on Volatile Composition and Sensory Properties of Stewed Pork

**DOI:** 10.3390/foods10010083

**Published:** 2021-01-04

**Authors:** Dong Han, Chun-Hui Zhang, Marie-Laure Fauconnier

**Affiliations:** 1 Laboratory of Agro-Products Processing, Ministry of Agriculture and Rural Affairs, Institute of Food Science and Technology, Chinese Academy of Agricultural Sciences, Beijing 100193, China; orange_1101@126.com; 2 Laboratory of Chemistry of Natural Molecules, Gembloux Agro-Bio Technology, University of Liege, 5030 Gembloux, Belgium; marie-laure.fauconnier@uliege.be

**Keywords:** stewed pork, seasoning formulations, volatile components, gas chromatography-mass spectrometry/olfactometry (GC-MS/O), two-dimensional gas chromatographic combined with time-of-fight mass spectrometry (GC × GC-TOFMS), principal component analysis (PCA)

## Abstract

The study aimed to investigate the influence of seasoning formulations (SP_1_: water; SP_2_: water and salt; SP_3_: water, salt and spices; SP_4_: water, salt, spices and soy sauce; SP_5_: water, salt, spices, soy sauce, sugar; SP_6_: water, salt, spices, soy sauce, sugar and cooking wine) on the volatile profiles and sensory evaluation of stewed pork. Volatile compounds were extracted using solid phase microextraction (SPME), then analysed by gas chromatography-mass spectrometry/olfactometry (GC-MS/O) and two-dimensional gas chromatographic combined with time-of-fight mass spectrometry (GC × GC-TOFMS). The results revealed that the most abundant volatile compounds, especially aldehydes, were presented in the stewed pork using SP_1_ and SP_2_. This indicated that the stewed pork with water and salt could promote lipid oxidation and amino acid degradation. As revealed by principal component analysis (PCA), the stewed pork samples with SP_3_ were located on the opposite side of that with SP_4_, SP_5_, and SP_6_ in the first and third principal component (PC1-PC3), which indicated that the overall flavour formed by adding spices was significantly different from that of adding soy sauce, sugar, and cooking wine. Sensory evaluation showed that stronger spicy, caramel, and soy sauce odour were present in samples SP_3_, SP_4_, SP_5_, and SP_6_. This study has indicated that the addition of food seasoning had a positive effect on flavour profiles of stewed pork, particularly for salt and spices.

## 1. Introduction

According to the report of State Statistical Bureau of China, pork production has increased to 54.0 million tons from 2014 to 2019, accounting for more than 60% of the meat production. Stewed pork, a representative Chinese style meat product, is appreciated by consumers in most regions of China due to its simple processing technique [1] and distinct flavour [2]. Usually, the stewed pork is often produced by stewing the fresh pork in water with various condiments and spices for a long time [3]. Owing to the differences in the dietary habits of domestic consumers, the manufacturer would add seasonings to make different flavoured stewed meat products to meet the clients’ needs. Some studies found that the seasonings created an enticing aroma during stewing and removed the undesirable odour in raw meat. Qin et al. [4] reported that a total of 37 volatile compounds were identified in stewed meat broths and the main volatile compounds, such as anethole, eucalptol, linalool, terpinen-4-ol, alpha-terpineol, and cedrol, may originate from star anise. More hexadecanal, octadecanal, and 9-octadecenal were present in soy sauce-stewed pork than in those water-boiled pork, and only 3,5-dimethyl-*trans*-1,2,4-trithiolane was detected in soy sauce-stewed pork [5]. It can be concluded that the addition of different food seasoning may have an important effect on the flavour formation of meat products. Although it seems reasonable that the flavour of stewed meat products would be associated with the addition of different food seasonings, there is currently little quantitative information available concerning the relationship between seasoning addition and the flavour of stewed meat.

To the best of our knowledge, salt, spices, soy sauce, sugar, and cooking wine are commonly used when cooking meat. Salt is a widely used additive in the meat industry due to its preservation and antimicrobial properties [6], and can accelerate lipid oxidation [7] to generate some lipid-related volatile compounds, such as linear-chain aldehydes and furans [8] during the heating treatment. The soy sauce and cooking wine contain many amino acids which may be the critical contributors to the characteristic flavour of the stewed pork [5,9]. Sugar, the flavour precursors to the Maillard reaction, usually plays an important role in the generation of aroma compounds of processed food. The studies had reported that the aldehydes, pyrazines and furans generated from Maillard reaction were considered as the key compounds of pot-stewed chicken meat products [10]. The formation of methyl aldehyde and pyrazine might come from the degradation of amino acids, which were partly associated with the reaction between reducing sugars and amino acids [11]. The spices are used as flavouring ingredient and natural antioxidants [12] in the processed meat products. For example, the addition of 0.5% garlic or onion before irradiation was effective in reducing lipid oxidation and provided some garlic/onion aromas for the cooked ground beef [13]. The addition of star anise changed the composition and proportion of volatile compounds, and imparted a spicy flavour to stewed chicken [14]. Although the important contribution of volatile compounds formed in common seasonings for meat products have been fully investigated, the sources and changes of flavour compounds produced by adding seasonings in the pork stewing process remain unknown.

A combination of gas chromatography-mass spectrometry (GC-MS) and olfactometric detector is used to connect the volatile compounds with sensory sniffing to more favourably and effectively identify the key odour-active compounds from numerous volatile constituents in the meat products, such as stewed pork broth [15] and braised pork [16]. Compared with gas chromatography-mass spectrometry/olfactometry (GC-MS/O), comprehensive two-dimensional gas chromatography-time-flight mass spectrometry (GC × GC-TOFMS) not only offered higher separation power, but also maintained more desirable sensitivity [17]. The GC × GC-TOFMS has been considered as a powerful technique for detailed profiling of the flavour profile of different food stuffs, such as braised chicken [10], dry-cured ham [18], and green teas [19]. Additionally, the key characteristic compounds and volatile profiles of the fresh and grilled eel were investigated by electronic nose (E-nose), GC-O, GC-MS, and GC × GC-TOFMS [17]. In summary, the combination of GC-MS/O and GC × GC-TOFMS is meaningful and facilitative for the identification of major flavour compounds and analysis of flavour fingerprints.

The purpose of this study was to characterize the volatile profiles of stewed pork via GC-MS/O combined with GC × GC-TOFMS, and evaluate the effect of the addition of water, salt, spices, soy sauce, sugar and cooking wine on the volatile compounds and sensory properties of stewed pork. Moreover, the differences and changes of flavour compounds in stewed pork with the increase of seasonings would be fully understood, and the relationship between sensory evaluation and odour-active compounds in different stewed pork samples may be also systematically explored. Thus, different seasoning formulae could be used as a valuable direction to produce the stewed pork with a desirable flavour for the consumers.

## 2. Materials and Methods

### 2.1. Sampling and Chemicals

A total of 42 pieces of hind leg muscles with an average weight of approximately 0.8–1.0 kg from Duroc × (Landrace × Yorkshire) pig breed (aged 5–6 months and with body weights of 90–95 kg) were obtained from Chuying Agro-Pastoral Group Co. Ltd. (Zhengzhou, Henan Province, China). All pigs were reared under the same conditions, provided with the same feed and slaughtered following routine abattoir procedures (stunned, exsanguinated, scalded, dehaired and eviscerate). The hind leg muscles from the carcasses were cut into strips (6.0 cm × 4.0 cm × 10.0 cm) after removing visible fat and connective tissues. Then these muscles were packed in low-density polyethylene bags and stored at −20 °C. The sampling procedures were approved by the Animal Care and Use Committee of the Institute of Food Science and Technology, Chinese Academy of Agricultural Sciences, and performed in accordance with animal welfare and ethics. The 2-methyl-3-heptanone (99%) and *n*-alkanes (C_7_–C_30_) of chromatographic grade were bought from Sigma-Aldrich (Shanghai, China).

### 2.2. Preparation of Stewed Pork Samples

#### 2.2.1. Preparation of Spice Bag

*Allium fistulosum* L. (45 g/kg) and *Zingiber officinale* Rosc. (30 g/kg) were chopped evenly into pieces with the knife. 9.0 g/kg *Piper nigrum* L., 1.2 g/kg *Cinnamomum cassia* Presl., 0.3 g/kg *Syringa oblata* Lindl., 1.0 g/kg *Myristica fragrans* Houtt., 0.5 g/kg *Glycyrrhiza uralensis* Fisch., 1.0 g/kg *lllicium verum* Hook.f., 0.3 g/kg *Cinnamomum wilsonii* Gamble., 0.2 g/kg *Elettaria cardamomum* (L.) Maton, 1.0 g/kg *Foeniculum vulgare* Mill., 0.2 g/kg *Amomum kravanh* Pierre ex Gagnep., 1.2 g/kg *Citrus reticulata Blanco*, 0.8 g/kg *Alpinia officinarum Hance*, 0.4 g/kg *Trifolium repens* L., 0.6 g/kg *Piper longum* Linn, 0.3 g/kg *Crataegus pinnatifida* Bunge, 0.4 g/kg *Zanthoxylum bungeanum* Maxim., 0.5 g/kg *Keampfera galangal* L., 0.5 g/kg *Amomum tsaoko* Crevost et Lemarie, and 1.2 g/kg *Angelica sinensis* (per kg of pork) were ground into powder by the high-speed grinder and then mixed. All the above materials were put into one spice bag for stewed pork.

#### 2.2.2. Seasoning Formulations and Stewed Pork

The pork strips were boiled in water for 10 min at 100 °C to remove blood, and then stewed for 45 min at 98 ± 2 °C with different seasoning formulations, finally soaked for 60 min. The flow diagram of the stewed pork is shown in Figure S1. For stewed pork, the ratio of pork strip to seasoning formulation was 1:2 (*w/v*). In this study, there were six seasoning formulations to which the following treatments were randomly assigned (formulation 1:2 L/kg water, formulation 2:2 L/kg water + 60 g/kg salt, formulation 3:2 L/kg water + 60 g/kg salt + one spice bag, formulation 4:2 L/kg water + 60 g/kg salt + one spice bag + 20 g/kg soy sauce, formulation 5:2 L/kg water + 60 g/kg salt + one spice bag + 20 g/kg soy sauce + 30 g/kg sugar, formulation 6:2 L/kg water + 60 g/kg salt + one spice bag + 20 g/kg soy sauce + 30 g/kg sugar + 30 g/kg cooking wine). The water and seasoning were weighed based on each kilogram of pork. The stewed pork with formulations 1, 2, 3, 4, 5, and 6 were marked stewed pork one (SP_1_), stewed pork two (SP_2_), stewed pork three (SP_3_), stewed pork four (SP_4_), stewed pork five (SP_5_) and stewed pork six (SP_6_), respectively. It is worth noting that SP_3_ was cooked with seasoning formulation that referred to aged brine according to the following procedure [20]. The first brine (fresh brine) was obtained by removing the spice bag and stewed pork, subsequently, supplementing water, salt and spice bag into fresh brine, the second brine was obtained by the same procedures. Aged brine was eventually produced based on this cyclic process until the eighth cycle. The processing methods of seasoning formulations of SP_4_, SP_5_, and SP_6_ were the same as that of SP_3_. Only difference was the composition of seasoning formulation, which contained more soy sauce, sugar and cooking wine in sequence than SP_3_. SP_1_ and SP_2_ were cooked with water and salt-water. Fresh pork (FP) without stewing was used as the control group. All pork samples were collected the vacuum bags and stored at −20 °C until used.

### 2.3. Extraction of Volatile Compounds

The extraction of volatiles from the stewed pork was carried out using the manual solid-phase micro-extraction (SPME) equipped with a 50/30 μm divinylbenzene/carboxen/polydimethylsiloxane (DVB/CAR/PDMS) fibre (Supelco, Inc., Bellefonte, PA, USA). Briefly, 5.0 g of the pork sample was weighed precisely and placed in a 40 mL headspace vial. Immediately after, 1 μL of 2-methyl-3-heptanone was added and sealed tightly with screw caps fitted with a Teflon/silicon septum. The vial was incubated in a thermostatic water bath at 60 °C for 20 min. The selected fibre was used to extract the volatile compounds in head space for 40 min at 60 °C. Upon completion, the fibre was inserted into the injection port (250 °C) of the GC instrument to desorb the analyses for 5 min.

### 2.4. GC-MS/O Analysis

The method was performed by the method of Han et al. [21] with minor modifications. The volatile compounds of stewed pork were analysed and identified by a GC-MS instrument (7890A-7000B, Agilent Technologise, Inc., Santa Clara, CA, USA) equipped with an olfactory detection port (Sniffer 9000; Brechbuhler, Schlieren, Switzerland). Capillary column DB-wax (30 m × 0.32 mm i.d., 0.25 µm film thickness; J & W Scientific, Inc., Folsom, CA, USA) was used with helium (purity of ≥99.999%) as the carrier gas at 1.2 mL/min flow rate. The front inlet temperature was 250 °C with a solvent delay of 4 min. The temperature program was as follows: Oven temperature was maintained at 40 °C for 3 min, ramped to 200 °C at a rate of 5 °C/min, then ramped to 240 °C at a rate of 10 °C/min with a 3 min final hold. The infector mode was splitless. The transfer line temperature and ion source temperature were kept 240 °C and 230 °C. Electro-impact mass spectra were generated at 70 eV, with *m/z* scan range from 50 to 400 amu. A sniffing port (Sniffer 9000) coupled to a GC-MS instrument was used for odour-active compound characterization. The effluent from the capillary column was split 1:1 (*v/v*) between the mass spectrometry detector and the olfactory detector port. The eight trained staff were utilized for the sniffing test on GC-O. The volatile compounds were initially identified by the National Institute of Standards and Technology (NIST) Mass Spectral Library (Version 2.0). Subsequently, the identified compounds were further confirmed based on a comparison of GC retention indices (RI) with authentic compounds. Qualitative analysis was also performed according to odour properties.

### 2.5. GC × GC-TOFMS Analysis

The GC × GC-TOFMS system consists of an Agilent 7890 gas chromatography (Agilent Technologies, Palo Alto, CA, USA) equipped with cold-jet modulator and time-of-fight mass spectrometer (TOFMS; LECO Pegasus 4D). The first column was DB-WAX (30 m × 0.25 mm i.d. × 0.25 μm film thickness) and the second column was DB-17HT (2 m × 0.1 mm i.d. × 0.1 μm film thickness). GC × GC conditions: the temperature of the injection port was 240 °C; helium (99.999%) flow, 1.0 mL/min; splitless injection; 6.0 s of modulation period; the column temperature program for the 1st D column: initial temperature was 40 °C and held for 1 min, increased to 220 °C at 3.0 °C/min, to 230 °C at 10 °C/min and held for 5 min; the column temperature program of the 2nd D column: initial temperature at 45 °C (held for 1 min), increased to 225 °C at 3.0 °C/min (held for 2.5 min), to 230 °C at 10 °C/min (held for 5 min). TOF-MS conditions: The ion source and transfer line to the mass spectrometer were maintained at 220 °C and 290 °C, respectively. The ionization potential of MS was 70 eV, the detector voltage was 1620 V, the scan range was 33 to 450 *m/z*, and the mass spectra data acquisition rate was 50 spectra/s. The identification of volatile compounds was based on the NIST 2014 library (Hewlett-Packard Co., Avondale, PA, USA), the mass spectral match factor ≥ 800 and similarity ≥ 1000.

### 2.6. Quantification of Volatile Compounds

The volatile compounds of stewed pork were semi-quantitated by the method of calibration with an internal standard (IS). The concentration of the volatile constituent was measured by the calibration curves of the GC-peak area and the amount ratios for the target analyst relative to 2-methyl-3-heptanone. The quantitative data of the identified compounds were obtained without considering the calibration factors, that is, the calibration factors were considered to be 1.00. The concentration of each compound was calculated as follows:

Conc(μgkg)=Peak area ratio(volatileIS) × 0.41 μg (IS)5.0 g (stewed pork)×1000

The odour activity value (OAV) of a compound was calculated as the ratio of its concentration in the stewed pork to its odour threshold in water. The equation is shown below:

$${\mathrm{OAV}}_{i}=\frac{C_{i}}{{\mathrm{OT}}_{i}}$$

where C_i_ is known as the concentration of the compound in the stewed pork and OT_i_ is the odour threshold in water. Compounds with OAV ≥ 1 are considered to be the main contributors to the total flavour.

### 2.7. Sensory Evaluation

Sensory evaluation was carried out by 8 trained panellists (4 females and 4 males, aged 25–35 years). All assessors were recruited from Chinese Academy of Agriculture Sciences, Beijing and had at least one year of experience in the sensory descriptive analysis of stewed meat products. In order to be more familiar with the flavour characteristics of stewed pork, the 12 weeks of training sessions (2 times per week and 2 h per session) were conducted by assessors. The panellist have descripted and defined the flavour attributes, reference standard and intensities (Supplementary Table S1). Samples were coded with three-digit randomized numbers and presented to the assessors at room temperature. Panellists selected five flavour attributes to be evaluated, namely, fatty odour, meaty odour, caramel odour, soy sauce odour and spicy odour. Each attribute was scored on a 10 cm non-structured line with anchor points at each end (0 = not perceivable, 10 = strongly perceivable) [22]. The mean value of sensory attributes was shown in the radar chart.

### 2.8. Statistical Analysis

The contents of volatile compounds and odour activity values (OAVs) of the odour-active compounds in stewed pork were presented as the mean ± standard deviation (SD). Significant differences were determined by one-way analysis of variance (ANOVA) and Duncan’s multiple range test at *p* < 0.05 of statistical product and service solutions (SPSS) software (v. 19.0, SPSS, Inc., Chicago, IL, USA). The PCA and PLS-DA were performed based on the mean OAVs using the software XLSTAT (2016) from Addinsoft (Barcelona, Spain). The data of odour-active compounds and sensory description in different stewed pork was also conducted with PLSR of the software XLSTAT (2016) from Addinsoft (Barcelona, Spain). The heat maps of the correlation data of PLS-DA and PLSR were conducted by R v3.2.2 (R Studio Team, 2012).

## 3. Results and Discussion

### 3.1. Analysis of Different Types of Volatile Components in Fresh and Stewed Pork

The kinds and content ratios of volatile components in all pork samples were shown in Table 1, the most abundant volatile compounds were aldehydes, followed by hydrocarbons, alcohols, heterocyclic and sulphur compounds, finally ketones, ethers, phenols, esters, and acids. These kinds of volatile compounds have been also found in cooked pork products [16,21]. In terms of the stewed pork with different seasoning formulations, the types and proportions of aldehydes, heterocyclic and sulphur-containing compounds increased significantly when the pork was stewed in water and salt (SP_1_ and SP_2_), while these compounds of pork samples treated with spices, soy sauce, sugar and cooking wine (SP_3_, SP_4_, SP_5_, and SP_6_) decreased slightly. This result indicated that a large amount of flavour compounds was formed during the thermal processing of pork, however some of them were inhibited due to the addition of edible condiments. Additionally, it has been reported that the peak area and percentage composition of aldehydes increased in stewed chicken with the addition of star anise, whereas that of Maillard reaction products decreased [14]. The numbers of alcohols and ketones had gradually increased with the addition of seasoning in pork samples, and the content ratios of hydrocarbons in samples SP_3_, SP_4_, SP_5_, and SP_6_ were much higher than those of samples from other treatment groups. These analyse concluded that the heat treatment and seasoning play the important role to the flavour of the cooked pork [23,24]. Furthermore, compared with compounds detected by GC-MS/O, more volatile compounds (e.g., aldehydes, alcohols, ketones, ethers, acids, heterocyclic, and sulphur-containing compounds) were identified by GC × GC-TOFMS, while some long-chain aldehydes and hydrocarbons were only detected using GC-MS/O (Supplementary Table S2). The above results have shown that the GC × GC-TOFMS combined with GC-MS/O could more comprehensively analyse the volatile profile in the fresh and stewed pork.

### 3.2. Volatile Compounds Profiling in the Fresh and Stewed Pork

A total of 139 volatile compounds of the fresh and stewed pork were extracted and identified by GC-MS/O and GC × GC-TOFMS, including 30 aldehydes, 23 alcohols, 19 ketones, 25 hydrocarbons, 13 esters, five ethers, three phenols, four acids, and 17 heterocyclic and sulphur-containing compounds (Table 2). Most of these compounds have been reported in several pork products [24,25]. Aldehydes are mainly derived from the lipid oxidation and degradation reaction, and Strecker degradation of amino acids [21,26], which are considered as the major contributions to the overall flavour of meat products [20,27] due to their lower odour thresholds. Among seven stewed pork samples, the content of aldehydes was the most abundant, with the total mean amounts of 719.6–3326.1 μg·kg^−1^. Obviously, the content of aldehydes in heated pork samples (SP_1_, SP_2_, SP_3_, SP_4_, SP_5_, and SP_6_) were significantly greater (*p* < 0.001) than those in the fresh pork. This indicated that the thermal processing of pork products plays a crucial role in the formation of aldehydes [23]. It can be also found that 15 compounds, namely, nine saturated aldehydes (butanal, pentanal, hexanal, heptanal, octanal, nonanal, decanal, pentadecanal and (*E*)-octadec-9-enal), four olefin aldehydes ((*E*)-oct-2-enal, (*E*)-non-2-enal, (*Z*)-dec-4-enal and (*E*)-dec-2-enal), as well as two aromatic aldehydes (benzaldehyde and benzeneacetaldehyde) were simultaneously identified in all stewed pork samples. The saturated and olefin aldehydes could be produced from the degradation of fatty acids [28] and the aromatic aldehydes were usually generated from Strecker reaction [29]. Among them, except for pentanal and benzeneacetaldehyde, the contents of other 13 compounds were significantly higher (*p* < 0.001) in SP_1_ and SP_2_ than those in SP_3_, SP_4_, SP_5_, and SP_6_. Moreover, (*Z*)-hept-2-enal, 2-butyloct-2-enal, and tertradecanal were detected only in SP_1_ and SP_2_. It was shown that the spices, soy sauce, sugar, and cooking wine may have an inhibitory effect on the production of some aldehydes in stewed pork. Moreover, 4-(1-methylethyl)-benzaldehyde, p-anisaldehyde and cinnamaldehyde, with mint and sweet aroma [20], could be generated from the Chinese different spices, including fennel, anise, and cinnamon, respectively.

Alcohols, with pleasant fruity and floral notes [18], are mainly derived from spices [30] and the oxidative decomposition of lipid [31]. For the identified alcohols, 1,8-cineole, pentan-1-ol, hexan-1-ol, oct-1-en-3-ol, heptan-1-ol, 2-etylhexan-1-ol, octan-1-ol and (*E*)-oct-2-en-1-ol could be detected in all pork samples. Among them, the linear alcohols (pentan-1-ol, hexan-1-ol, heptan-1-ol, octan-1-ol and (*E*)-oct-2-en-1-ol) and branched alcohols (oct-1-en-3-ol and 2-etylhexan-1-ol) were the main lipid oxidation products. Meanwhile, the contents of these compounds were significantly higher (*p* < 0.01) in SP_1_ and SP_2_ than those in FP, SP_3_, SP_4_, SP_5_ and SP_6_. The 1,8-cineole, linalool, terpinen-4-ol, terpineol, (4R,6R)-2-undecen-1-ol and cis-geraniol increased significantly (*p* < 0.001) in SP_3_ as the spices were added in the stewed pork and greatly reduced in SP_4_, SP_5_ and SP_6_. This result shows that these above compounds were mainly formed from aged brine (spices, soy sauce, sugar and cooking wine).

Ketones are often considered to have a great influence on the aroma of meat and meat products since they are presented in large amounts and exhibited specific aroma in food [32]. As shown in Table 2, 7 ketones such as 6-methylhept-5-en-2-one, 4-isopropyl-2-cyclohexenone, piperitone, carvone, 4-phenylbutan-2-one, 4-methoxyphenylacetone and xanthoxylin were detected in the spice, soy sauce, sugar and cooking wine processed samples (SP_3_, SP_4_, SP_5_ and SP_6_), and they were not detected in fresh pork (FP), stewed pork with water or salt (SP_1_ or SP_2_). It indicated that more ketone compounds were formed due to the addition of food seasoning, which provided a richer fruity and nutty aroma [25] for the overall pork flavour. Pham et al. [33] reported that the methyl ketones were considered as the precursors to the fatty aromas related to cooked meat, which could be formed by the oxidative degradation of fatty acids [34]. In this study, the methyl ketones (e.g., heptan-2-one, octan-2-one, pentane-2,3-dione, octane-2,3-dione, 3-octen-2-one, (*E,E*)-3,5-octadien-2-one, 6-methyl-3,5-heptadiene-2-one and acetophenone) were originated from the oxidation of lipids during heating.

There were few hydrocarbons detected in the fresh pork, however more hydrocarbons could be formed by added water, salt, soy sauce, sugar and cooking wine during the pork processing. All hydrocarbons could be divided into aromatic hydrocarbons and aliphatic hydrocarbons. Among them, 16 aromatic hydrocarbons had been identified in all stewed pork samples, and toluene, ethylbenzene, 1,3-dimethylbenzene, o-xylene and styrene were presented as common compounds. The production of toluene and ethylbenzene primarily come from amino acid degradation. This result was consistent with that reported by Olivares et al. [11]. It was also found that nine aliphatic hydrocarbons were detected in stewed pork samples (SP_1_, SP_2_, SP_3_, SP_4_, SP_5_ and SP_6_). Previous study has shown that aliphatic hydrocarbons had a limited influence on aroma perception due to their high threshold values [35] and raised mainly from lipid oxidation [27]. Additionally, phellandrene, (Z)-3,7-dimethyl-1,3,6-octatriene, o-cymene, m-cymene, 1-methylindan, and longifolene could be formed from the added spices and soy sauce during the processing.

Ester compounds could be formed by the esterification of alcohols and carboxylic acids in the meat products [2]. The contribution of esters to the aroma of pork products depends on the length of their chain [27]. A total of 13 esters were identified in all stewed pork samples, where short-chain esters, such as ethyl acetate and ethenyl acetate, had fruity notes. While long-chain esters like isoamyl isobutyrate, hexyl butanoate, hexyl acetate and hexyl butanoate possessed a slight fatty odour [36]. In addition, when the salt was added, the relative content of esters was significantly increased (*p* < 0.05). This reason might be that the salt of meat products favoured the formation of ester compounds. For ether and phenol compounds, the anethole was detected in all pork samples and the methyleugenol, elemicin, myristicin, eugenol and trans-isoeugenol were found in SP_3_, SP_4_, SP_5_ and SP_6_. Moreover, the ethers and phenols except for phenol were from the spices, and the acids (butanoic acid, pentanoic acid, and octanoic acid) come from the fresh pork.

Heterocyclic and sulphur-containing compounds are the important contributions to the formation of flavour in meat products [37]. As shown in Table 2, 17 heterocyclic compounds (furans, pyridines and pyrroles) and sulphur-containing compounds were detected in all pork samples. The 2-pentylfuran and safrole were derived from linoleic acid autoxidation [38] and spices (nutmeg, anise and ginger), respectively. The 2-pentylfuran is often used as an important indicator of the degree of oxidation of meat product. The contents of 2-pentylfuran in SP_1_ and SP_2_ were significantly higher (*p* < 0.001) than that in SP_3_, SP_4_, SP_5_, and SP_6_, indicating that the stewed pork with only water and salt had a greater effect on lipid oxidation. It has been reported that a large number of furans, pyridines, pyrroles and sulphur-containing compounds could be produced by Maillard reaction and amino acid degradation during cooking [15,23]. In our study, 3-(4-methyl-3-pentenyl)-furan, furfural, 2-furanmethanol, pyridine, 2-acetylpyrrole and dimethyl trisulfide displayed significantly higher levels (*p* < 0.01) in SP_5_ and SP_6_ than those in other groups, which indicated that the addition of sugar and cooking wine could promote the Maillard reaction. For sulphur-containing compounds, methanethiol and dimethyl disulphide from sulphur-containing amino acid degradation were significantly lower (*p* < 0.001) in SP_1_ than that in SP_2_, which indicated that salt-treated stewed pork was more conducive to the production of sulphur-containing compounds. This result was consistent with that reported by Liu et al. [39] who found that the levels of sulphur-containing compounds in Nanjing water-boiled salted duck were markedly higher than those in control samples. Regarding 2-acetylthiazole, 2-thiophenecarboxaldehyde and benzothiazole originated from Maillard reaction, and they contributed to roasted, caramel, and meaty notes [40] for the overall aroma of stewed pork.

**Figure 1:**
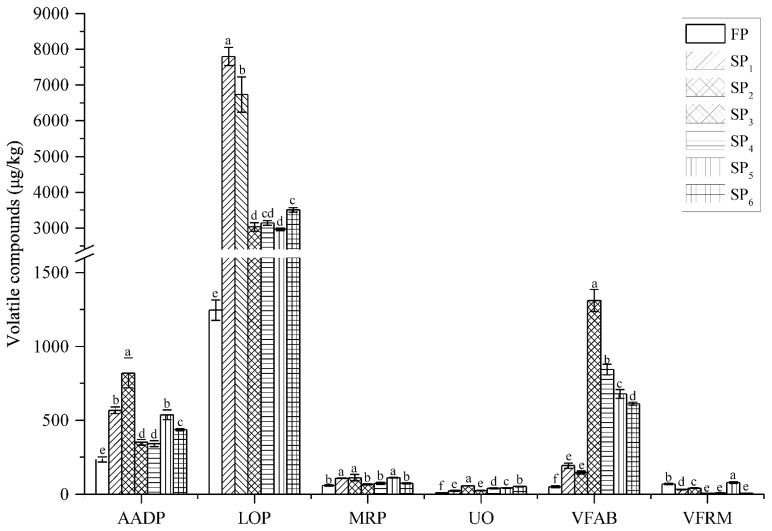
Concentration of volatile compounds according to possible origins of the fresh and stewed pork. Different letters are significantly different (*p* < 0.05) in each pork treatment group. FP, fresh pork; SP_1_, boiled pork with water; SP_2_, cooked pork with water and salt; SP_3_, stewed pork with water, salt and spices; SP_4_, stewed pork with water, salt, spices and soy sauce; SP_5_, stewed pork with water, salt, spices, soy sauce and sugar; SP_6_, stewed pork with water, salt, spices, soy sauce, sugar and cooking wine. LOP, Lipid oxidation products; AADP, Amino acid degradation products; MRP, Maillard reaction products; VFAB, Volatiles from aged brine; VFRM, Volatiles from raw meat; UO, Unknown origin.

The concentration of volatile compounds according to possible origins of the fresh and stewed pork are presented in Figure 1. It was found that the lipid oxidation, aged brine and amino acid degradation were the important origins of volatile compounds in all stewed pork attributed to their contribution to more aroma of the pork samples. For the lipid oxidation and amino acid degradation, their concentrations were highest in SP_1_ and SP_2_, followed by SP_3_, SP_4_, SP_5_ and SP_6_, finally FP. This indicated that heat-treated pork with water and salt would facilitate lipid oxidation and amino acid degradation to produce more volatiles, while there was an inhibitory effect on heat-treated pork with aged brine, especially for spices. Compared with volatile compounds from the aged brine in SP_3_, SP_4_, SP_5_ and SP_6_, they were significantly higher (*p* < 0.05) than those in SP_1_ and SP_2_, this may be due to the addition of food condiments (spices, soy sauce, sugar, and cooking wine) in stewed pork.

### 3.3. Odour-Active Compounds Analysis of the Fresh and Stewed Pork

To evaluate the contributions of volatile compounds to overall flavour of the fresh and stewed pork, the OAVs of these compounds were determined by dividing the concentration of the compound by its odour threshold in water. As can be seen from Table 3, a total of 29 odour-active compounds with OAVs greater than 1 were selected from 139 volatile compounds, including 14 aldehydes, four alcohols, three ketones, one hydrocarbon, one ester, two ethers, one phenol, thre furans, N- or S-containing compounds. Seven of them with relatively high OAVs were detected in all stewed pork samples: hexanal (OAV at 44.1–158.5), heptanal (OAV at 10.5–53.4), octanal (OAV at 72.2–329.9), nonanal (OAV at 110.3–1475.5), oct-1-en-3-ol (OAV at 13.9–68.5), 2-pentylfuran (OAV at 39.0–178.6) and methanethiol (OAV at 31.3–80.1). These compounds were known as the key odour-active compounds due to their significant contributions to the integral flavour. Furthermore, it was found that the OAVs of hexanal, heptanal, octanal, nonanal, and decanal increased significantly (*p <* 0.01) in SP_1_ and SP_2_.

Statistical analysis showed that the total OAVs of odour-active compounds of SP_1_ and SP_2_ were significantly higher (*p* < 0.001) than those of FP and SP_3_, SP_4_, SP_5_ and SP_6_. Linear aldehydes like pentanal, hexanal, heptanal, octanal, nonanal and decanal have been reported to be generated from lipid oxidation [27]. Moreover, these aldehyde compounds could be detected in different processing methods and may contribute grassy, fatty and fruity notes to overall aroma of the pork samples. Unsaturated aldehydes such as (*Z*)-hept-2-enal, (*E*)-oct-2-enal, (*E*)-non-2-enal, (*E*)-dec-2-enal, (*E,E*)-2,4-nonadienal, undec-2-enal, and (*E,E*)-2,4-decadienal are degradation products of linoleate and linolenate hydroperoxides [41]. Among them, there was no significant difference in undecen-2-al in FP, SP1, and SP2 (*p* > 0.05), indicating that heating and salt treatment had no effect on the formation of undecen-2-al. On the other hand, the rest of the olefin aldehydes have relatively higher OAVs in SP_1_ and SP_2_. This showed that SP_1_ and SP_2_ could promote the increase of some unsaturated aldehydes. Benzeneacetaldehyde, with honey and sweet notes, is a well-known aroma component formed from Maillard reaction of phenylalanine [42] and the OAV in SP_5_ were significantly higher (*p* < 0.001) than that in other samples. 1,8-Cineole, anethole and estragole, with mint and aniseed flavour, were the most abundant in SP_3_. The OAVs of oct-1-en-3-ol, (*E*)-oct-2-en-1-ol, butane-2,3-dione, octane-2,3-dione and 2-pentylfuran were the highest in SP_1_ and SP_2_, followed by SP_3_, SP_4,_ SP_5_ and SP_6_, of which oct-1-en-3-ol and octane-2,3-dione was shown to be richer in SP_2_ than that in SP_1_. Linalool and eugenol were found immediately when the spices were added to the cooked pork, which may be due to the flavour of the star anise itself. Dimethyl trisulfide, with fish and cabbage notes, was considered as the main sulphur-compound in SP_1_, SP_4_, SP_5_, and SP_6_.

### 3.4. PCA and PLS-DA Analysis of Odour-Active Compounds

**Figure 2:**
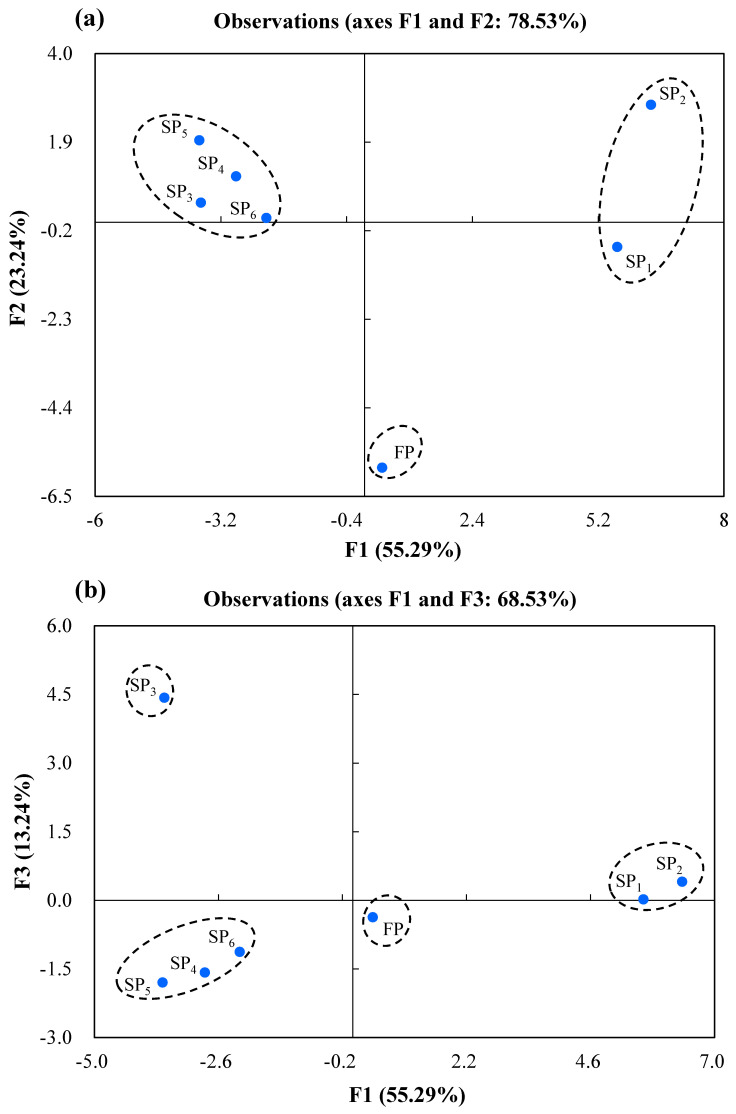
Score plots of PCA of the fresh and stewed pork. (**a**) PC1 plotted against PC2 and (**b**) PC1 against PC3. FP, fresh pork; SP_1_, stewed pork with water; SP_2_: stewed pork with water and salt; SP_3_: stewed pork with water, salt and spices; SP_4_: stewed pork with water, salt, spices and soy sauce; SP_5_: stewed pork with water, salt, spices, soy sauce and sugar; SP_6_: stewed pork with water, salt, spices, soy sauce, sugar and cooking wine.

In order to clarify the differences in aroma profile of the fresh and stewed pork, a principal component analysis (PCA) was performed and showed in Figure 2a,b. The first principal component (PC1) explained 55.17%, the second principal component (PC2) explained 22.85% and the third principal component (PC3) explained 13.22% of the variations. The first three principal components (PCs) accounted for 91.24% of the total variance and were enough to explain the maximum variation in all original data of the pork samples. As can be seen in Figure 2a, the PC1 and PC2 showed a clear-cut separation of the samples into three major groups. Among them, the sample dot of FP was located in the fourth quadrant, and sample dots representing SP_3_, SP_4_, SP_5_, and SP_6_ were located in the second quadrant and could be recognized as one cluster, while sample dots of SP_1_ and SP_2_ located in the first and fourth quadrant were considered as a group because of their relatively closer distance. As shown in Figure 2b, the fresh and stewed pork samples were divided into four groups in PC1 and PC3, that is, group I: FP; group II: SP_1_ and SP_2_; group III: SP_3_; group IV: SP_4_, SP_5,_ and SP_6_. The four group of sample points were located in the different quadrants indicating that the overall aroma of each group of samples were different. Moreover, SP_4_, SP_5_, and SP_6_ samples was close and located in the third quadrant. It can be concluded that the overall flavour of SP_4,_ SP_5_, and SP_6_ was similar each other. Similarly, so was SP_1_ and SP_2_. It can be also found that the flavour of samples SP_4,_ SP_5_ and SP_6_ was significantly different from that of sample SP_3_ in Figure 2a,b. This showed that the flavour compounds of stewed pork were also affected by soy sauce, sugar, and cooking wine.

**Figure 3:**
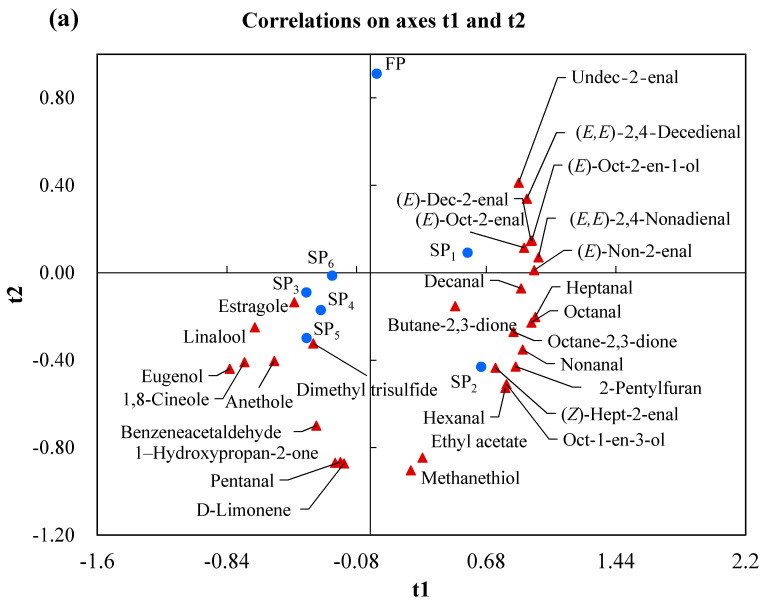

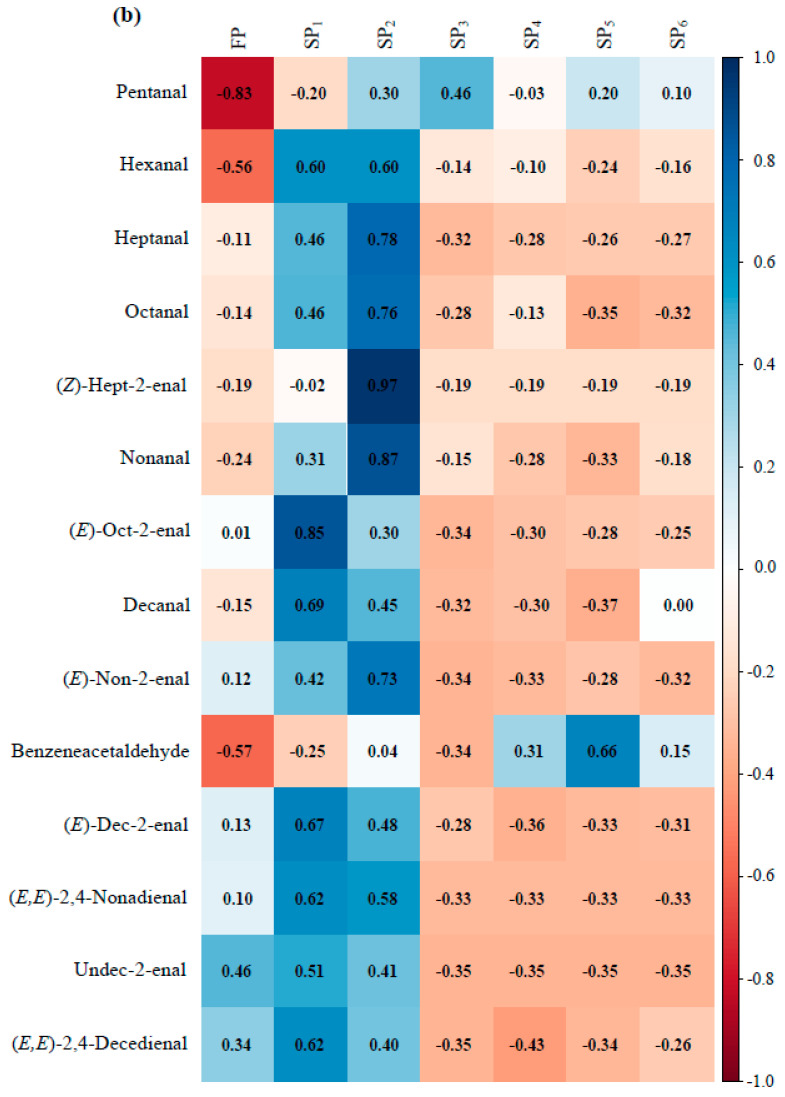

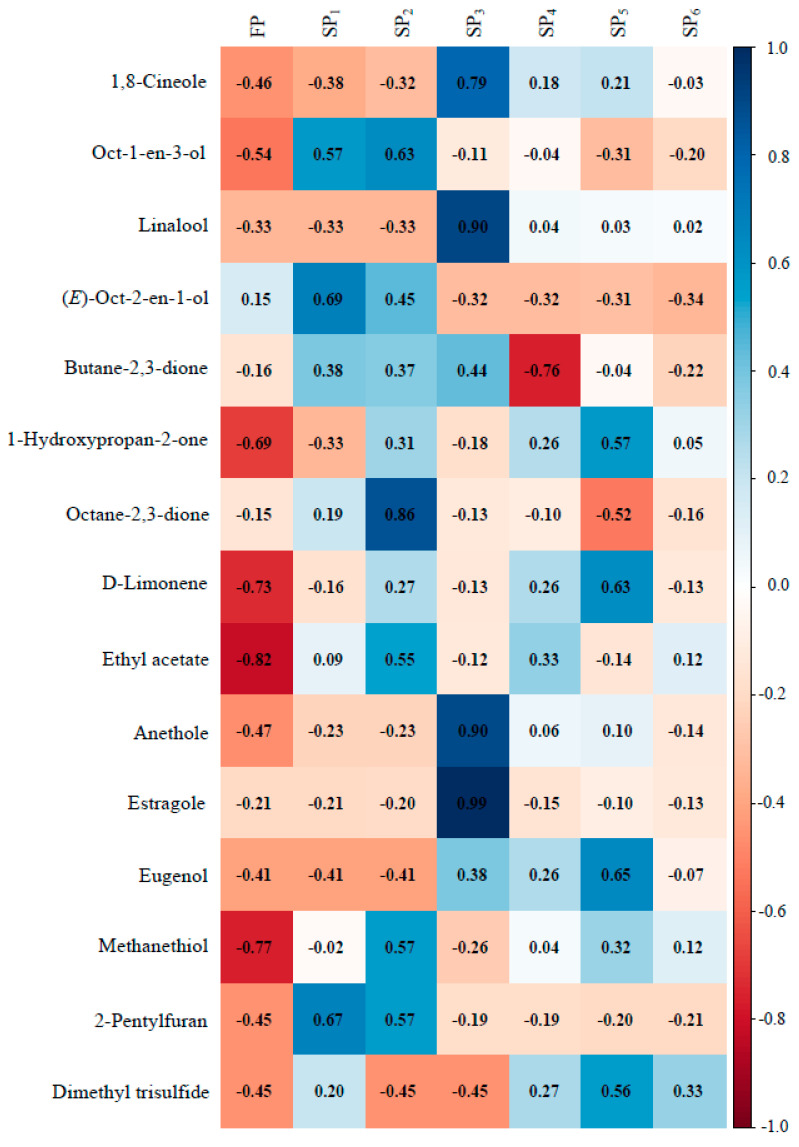
(**a**) Loading biplot of t1 and t2 of the model performed after PLS-DA of the volatile compounds in different pork samples (R^2^X = 0.968, R^2^Y = 0.818 and Q^2^ = 0.628). (**b**) Heat map of the correlations between volatile compounds and the pork samples. FP, fresh pork; SP_1_, stewed pork with water; SP_2_: stewed pork with water and salt; SP_3_: stewed pork with water, salt and spices; SP_4_: stewed pork with water, salt, spices and soy sauce; SP_5_: stewed pork with water, salt, spices, soy sauce and sugar; SP_6_: stewed pork with water, salt, spices, soy sauce, sugar and cooking wine. The blue circle dots represent the fresh and stewed pork and the red triangle dots represent the odour-active compounds.

Apart from PCA, the supervised PLS-DA was performed to evaluated the differences of volatile compounds of the stewed pork. As shown in Figure 3a, except for SP_3_, SP_4,_ SP_5_ and SP_6_, only the separation was observed for FP, SP_1_, and SP_2_ (R^2^X = 0.968, R^2^Y = 0.818 and Q^2^ = 0.628). SP_3_, SP_4,_ SP_5_, and SP_6_ were located on the negative side of axis 1, whereas FP, SP_1_, and SP_2_ were founds the positive side of axis1, SP_1_ and SP_2_ were close each other. Obviously, the different stewed pork samples were separated into three group (SP_3_-SP_4_-SP_5_-SP_6_, SP_1_-SP_2_, FP). It could also be concluded that the overall flavour of SP_3_-SP_4_-SP_5_-SP_6_, SP_1_-SP_2_ and FP were greatly different, and each group samples possessed the similar flavour profiles. The result is consistent with the PCA analysis (Figure 2a). In addition, to identify the most discriminative volatiles contributing to the observed the fresh and stewed pork samples, variable identification (VID) coefficients were calculated (Figure 3b). Volatiles with VID ≥ |0.80| discriminating FP, SP_1_, SP_2_ and SP_3_ were predominantly aldehyde compounds (pentanal, (*Z*)-hept-2-enal, nonanal and (*E*)-oct-2-enal) and related volatiles (linalool, octane-2,3-dione, ethyl acetate, anethole and estragole). Moreover, no compound with VID ≥ |0.80| was found discriminating SP_4_, SP_5_, and SP_6_. As shown in Figure 3a,b, it can be observed that pentanal, 1-hydroxypropan-2-one, D-limonene, ethyl acetate and methanethiol were not only on the opposite side of samples FP, but also strongly and negatively correlated with it (−0.83 ≤ r ≤ −0.69), while the remaining 24 odour-active compounds (−0.57 ≤ r ≤ 0.46) had a low correlation with it. Most odour-active compounds, such as hexanal, heptanal, octanal, (*Z*)-hept-2-enal, nonanal, (*E*)-oct-2-enal, decanal, (*E*)-non-2-enal, (*E*)-dec-2-enal, (*E,E*)-2,4-nonadienal, (*E,E*)-2,4-decedienal, oct-1-en-3-ol, (*E*)-oct-2-en-1-ol, octane-2,3-dione, and 2-pentylfuran, were close to SP_1_ and SP_2_ on the right side of t1, and the strong and positive correlation (0.60 ≤ r ≤ 0.97) were showed. Moreover, SP_2_ induced an increase in the correlation coefficients of heptanal, octanal, (*Z*)-hept-2-enal, (*E*)-non-2-enal, oct-1-en-3-ol and octane-2,3-dione indicating that the addition of salt during the processing of stewed pork was beneficial to the formation of these compounds. For SP_3_, SP_4,_ SP_5_ and SP_6_, benzeneacetaldehyde, 1,8-cineole, linalool, D-limonene, anethole, estragole and eugenol had the high and positive correlation coefficients (0.63 ≤ r ≤ 0.99) on the left side of t1, only butane-2,3-dione had the high and negative correlation coefficient (r = −0.76). Because 1,8-cineole, linalool, anethole, and estragole had a higher correlation coefficient (0.79 ≤ r ≤ 0.99) with SP_3_ and a lower correlation coefficient (−0.15 ≤ r ≤ 0.21) with SP_4,_ SP_5_, and SP_6_, this suggests that they may be potential flavour markers to distinguish SP_3_ and SP_4,_ SP_5_ and SP_6_.

### 3.5. Descriptive Sensory Analysis

**Figure 4:**
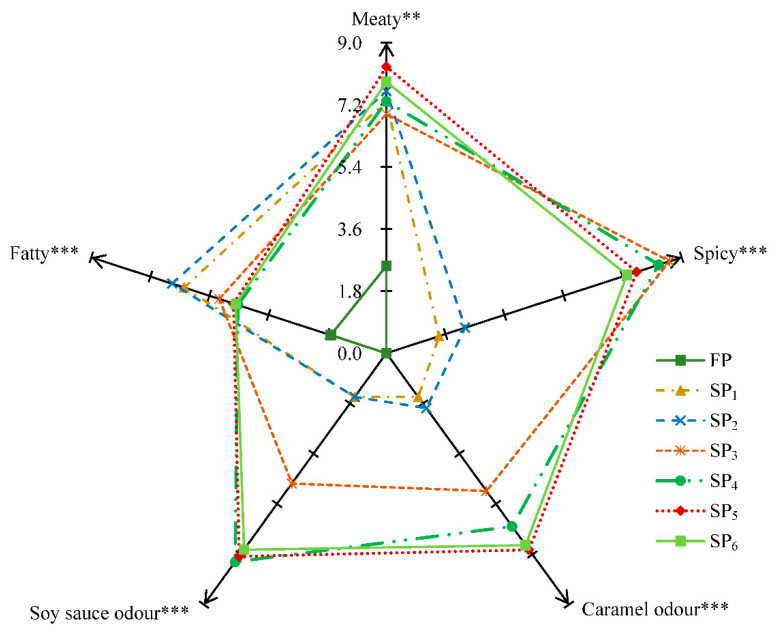
The odour sensory profiles of the fresh and stewed pork. Asterisks indicate significant (** *p* ≤ 0.01; *** *p* ≤ 0.001) differences of means in descriptor intensities. FP, fresh pork; SP1, stewed pork with water; SP2: stewed pork in water and salt; SP3: stewed pork in water, salt and spices; SP4: stewed pork in water, salt, spices and soy sauce; SP5: stewed pork in water, salt, spices, soy sauce and sugar; SP6: stewed pork in water, salt, spices, soy sauce, sugar and cooking wine.

To describe the differences of odour profiles of seven pork samples, the flavour sensory evaluation was performed using the five representative descriptors, namely, “meaty”, “spicy”, “caramel”, “soy sauce” and “fatty”. As can be seen from Figure 4. Significant differences (*p* ≤ 0.01) were found among five odour attributes of the pork samples. The intensities of fatty notes in SP_1_ and SP_2_ were highest, followed by SP_3_, SP_4,_ SP_5_, SP_6_ and FP. As described by the panellists from GC-MS/O, hexanal, heptanal, octanal, (*Z*)-hept-2-enal, nonanal, (*E*)-oct-2-enal, decanal, (*E*)-non-2-enal, (*E*)-dec-2-enal, (*E,E*)-2,4-nonadienal, (*E,E*)-2,4-decedienal and 2-pentylfuran might be closely related to the fatty odour. This result agreed with the result of Figure 3b. The meaty and caramel odour of SP_5_ and SP_6_ had the highest score in all samples, which could be mainly attributed to furans and N-containing compounds such as 3-(4-methyl-3-pentenyl)-furan, furfural, 2-furanmethanol, pyridine, and 2-acetylpryrrole. In addition, the strong spicy and soy sauce smell was presented in SP_3_ and SP_5_, respectively.

### 3.6. Relationship between Sensory Evaluation and Odour-Active Compounds

**Figure 5:**
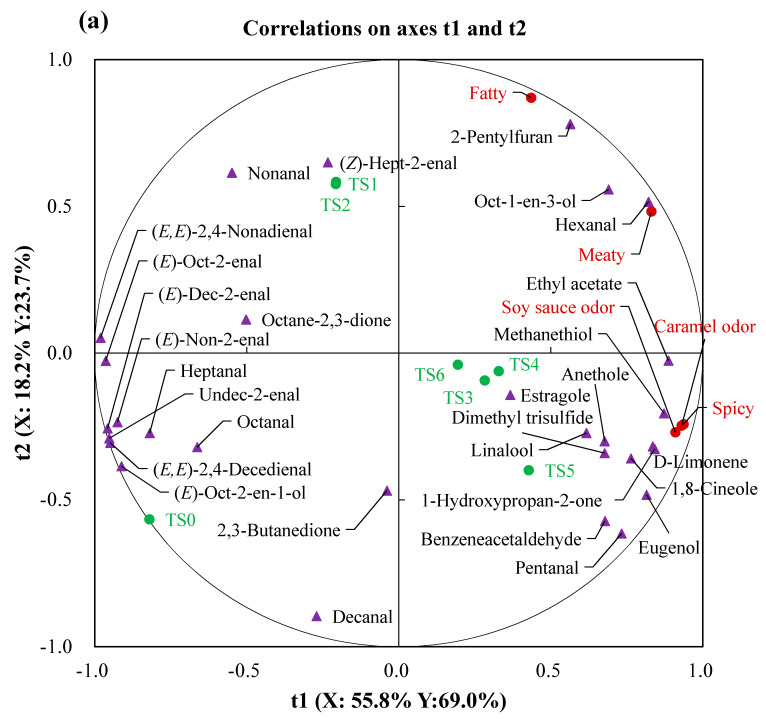

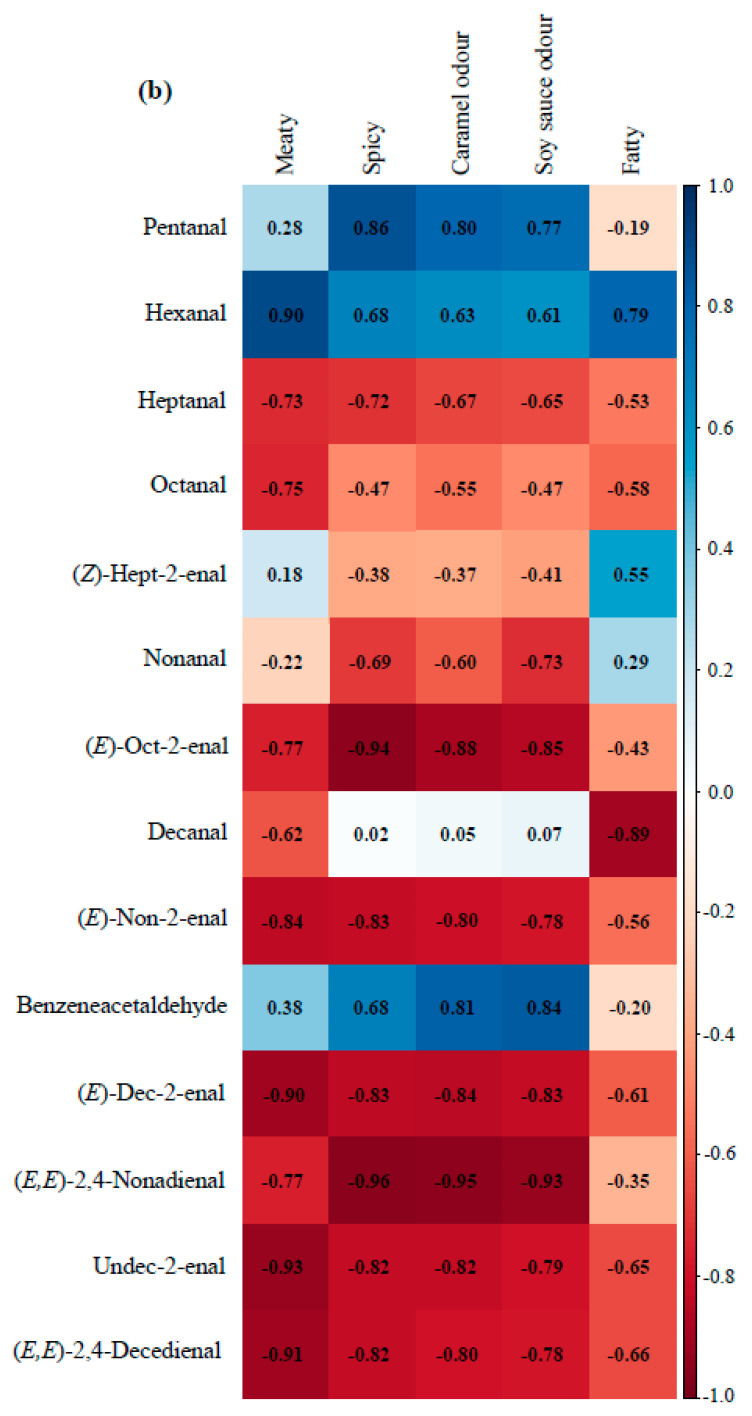

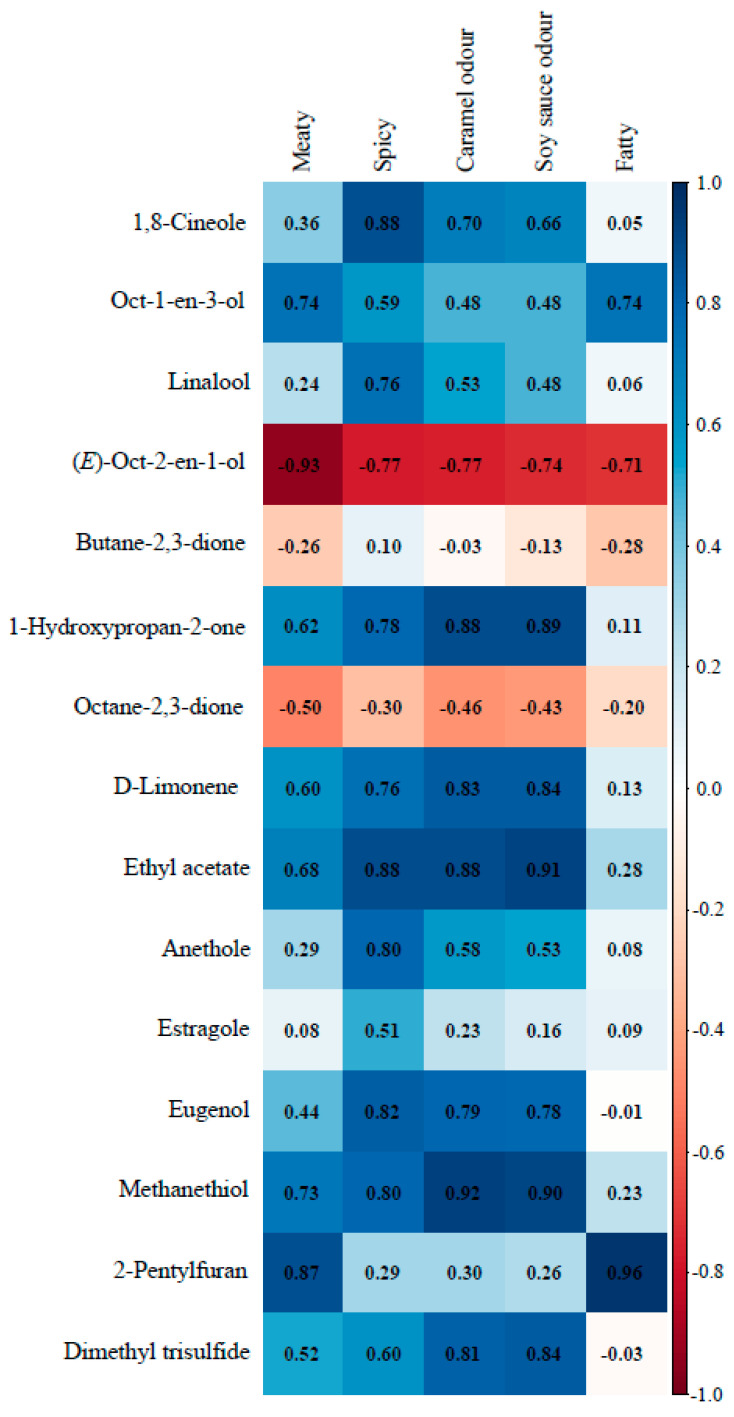
(**a**) PLSR loading for the odour attributes (Y variables) and the odour-active compounds of the fresh and stewed pork (X variables). (**b**) Heat map illustrating the Pearson correlation between descriptor intensities and proportion of OAVs of the odour-active compounds calculated by PLSR. The green circle dots represent 7 pork samples, the red circle dots represent 5 aroma attributes and the purple triangle dots represent 29 odour-active compounds.

PLSR was employed to establish the relationship between the five sensory descriptors of the fresh and stewed pork and the odour-active compounds analysed by GC-MS/O and GC × GC-TOFMS, and the correlation coefficient between them was expressed in the heat map. As shown in Figure 5a, most of the *X*-matrix (contribution ratios of the odour-active compounds) and *Y*-matrix (intensities of the sensory attributes) are loaded around the circle (*r*^2^ = 100%, *r*^2^ = represent the degree of interpretation). The model quality (Q^2^ = 0.846) ≥ 0.50 indicated that they were well explained by the PLSR model. The first two components explained 74.0% of *X*-matrix and 92.7% of *Y*-matrix. The dots corresponding to sample SP_1_ and SP_2_ had overlap in the second quadrant, and the samples points of SP_3_, SP_4_, SP_5_ and SP_6_ were close in the fourth quadrant, and as well as FP was found in the third quadrant. So, the fresh and stewed pork samples can be divided into three group and this result was consistent with previous PCA plots (Figure 2a). According to Figure 5a,b, it can be observed that SP_3_, SP_4_, SP_5_, and SP_6_ were characterized by soy sauce, caramel and spicy odour because of their short distance, and the three aroma attributes aforementioned were positively correlated with pentanal, benzeneacetaldehyde, 1,8-cineole, 1-hydroxypropan-2-one, D-limonene, ethyl acetate, eugenol, methanethiol, and dimethyl trisulfide with a high correlation coefficient (0.60 ≤ r ≤ 0.92). On the contrary, the soy sauce, caramel and spicy notes located in the right side of the loading plot were strongly and negatively correlated with some aldehydes (heptanal, nonanal, (*E*)-oct-2-enal, (*E*)-non-2-enal, (*E*)-dec-2-enal, (*E,E*)-2,4-nonadienal, undec-2-enal and (*E,E*)-2,4-nonadienal) and unsaturated alcohols like (*E*)-oct-2-en-1-ol. SP_1_ and SP_2_ on the upper left side of loading plot were mainly descriptive fatty and meaty odour, which were in accordance with the descriptive sensory analysis. These two attributes were highly associated with hexanal, oct-1-en-3-ol and 2-pentylfuruan. Moreover, FP was located far from these flavour attributions and most volatiles, which indicated that there was not the unique flavour of the fresh pork.

## 4. Conclusions

The volatile profile of fresh and stewed pork was more fully characterized using GC-MS/O and GC × GC-TOFMS analysis. A total of 139 volatile compounds were identified from all stewed pork and seven of which were confirmed as the key odour-active compounds, namely hexanal, heptanal, octanal, nonanal, oct-1-en-3-ol, 2-pentylfuran, and methanethiol. The fresh and stewed pork could be classified to four groups (FP, SP_1_-SP_2_, SP_3_, and SP_4_-SP_5_-SP_6_) by PCA based on the odour-active compounds, which showed that volatile profile of pork stewed with water and salt possessed the similar flavour, and the flavour composition of stewed pork with soy sauce, sugar, and cooking wine was not significantly different. However, there were significant differences in the overall flavour between pork samples of different groups. It can be concluded that the seasoning played a vital role in the flavour contribution of stewed pork, especially salt and spices. Additionally, the results of PLSR indicated that samples SP_3_, SP_4_, SP_5_, and SP_6_ were positively correlated with soy sauce, caramel, and spicy notes. On the contrary, soy sauce, caramel, and spicy odours were strongly and negatively correlated with samples SP_1_ and SP_2_, which was consistent with the result of sensory evaluation.

## Figures and Tables

**Table 1:** The comparison of kinds and content ratios of volatile compounds by gas chromatography-mass spectrometry/olfactometry (GC-MS/O) and two-dimensional gas chromatographic combined with time-of-fight mass spectrometry (GC × GC-TOFMS).

	Group	GC-MS/O							GC × GC-TOFMS						
		FP	SP_1_	SP_2_	SP_3_	SP_4_	SP_5_	SP_6_	FP	SP_1_	SP_2_	SP_3_	SP_4_	SP_5_	SP_6_
Quantities	Aldehydes	5	12	12	11	11	11	11	20	22	22	20	19	21	22
	Alcohols	3	5	5	6	7	7	8	14	13	16	17	19	16	18
	Ketones	0	0	0	3	3	3	3	8	10	12	13	11	14	13
	Hydrocarbons	6	11	13	11	13	13	14	6	11	12	18	13	15	13
	Esters	0	0	0	0	0	0	0	1	8	10	11	10	11	11
	Ethers	1	2	1	3	4	4	4	1	1	2	5	5	5	5
	Phenols	0	0	0	2	2	2	2	1	0	0	2	2	2	2
	Acids	0	0	0	0	0	0	0	3	0	2	2	2	2	2
	Heterocyclic and sulfur compounds	0	2	2	1	1	1	1	3	7	10	12	13	13	13
	Total	15	32	33	37	41	41	43	57	72	86	100	94	99	99
Ratios (%)	Aldehydes	37.8	66.8	63.7	34.9	40.2	37.8	43.2	43.1	30.6	38.9	20.0	17.9	18.5	19.9
	Alcohols	9.1	11.8	12.1	26.3	21.1	21.4	18.5	13.5	13.3	6.2	15.8	7.8	8.1	6.6
	Ketones	0.0	0.0	0.0	6.0	3.6	4.6	3.2	23.5	9.0	16.5	11.1	11.4	7.3	11.8
	Hydrocarbons	48.4	12.3	16.7	18.9	24.6	23.7	25.4	16.8	29.1	19.7	40.7	50.1	47.3	48.9
	Esters	0.0	0.0	0.0	0.0	0.0	0.0	0.0	0.1	2.8	5.2	2.5	3.7	9.1	4.4
	Ethers	4.8	1.2	1.0	6.2	3.7	4.6	3.6	0.2	0.2	0.2	2.4	0.8	0.9	0.5
	Phenols	0.0	0.0	0.0	2.9	3.8	4.5	2.7	0.01	0.0	0.0	0.5	0.5	0.7	0.2
	Acids	0.0	0.0	0.0	0.0	0.0	0.0	0.0	2.7	0.3	0.5	0.4	0.3	0.6	0.5
	Heterocyclic and sulfur compounds	0.0	7.9	6.4	4.9	3.0	3.3	3.4	0.1	14.7	12.9	6.5	7.5	7.5	7.0
	Total	100.0	100.0	100.0	100.0	100.0	100.0	100.0	100.0	100.0	100.0	100.0	100.0	100.0	100.0

Note: FP, fresh pork; SP1, stewed pork with water; SP2: stewed pork with water and salt; SP3: stewed pork with water, salt and spices; SP4: stewed pork with water, salt, spices and soy sauce; SP5: stewed pork with water, salt, spices, soy sauce and sugar; SP6: stewed pork with water, salt, spices, soy sauce, sugar and cooking wine.

**Table 2:** Concentrations and origin of volatile compounds of the fresh and stewed pork.

No.	Compounds	FP	SP_1_	SP_2_	SP_3_	SP_4_	SP_5_	SP_6_	*p* Value	Origin ^1^
	**Aldehydes (30)**	719.6 ± 36.8 ^d^	2478.5 ± 104.6 ^b^	3326.1 ± 219.3 ^a^	959.1 ± 1.9 ^c^	795.9 ± 7.8 ^cd^	815.1 ± 25.8 ^cd^	934.2 ± 44.1 ^c^	0.000	
1	Acetaldehyde	1.7 ± 0.2 ^a^	N.D.	N.D.	N.D.	N.D.	1.7 ± 0.3 ^a^	0.9 ± 0.1 ^b^	0.011	AADP
2	Propanal	N.D.	N.D.	97.9 ± 15.0 ^a^	N.D.	16.1 ± 3.2 ^b^	25.5 ± 1.7 ^b^	29.3 ± 2.4 ^b^	0.000	AADP
3	Butanal	0.5 ± 0.2 ^d^	3.7 ± 0.8 ^b^	19.0 ± 1.5 ^a^	2.1 ± 0.9 ^c^	3.5 ± 0.6 ^b^	2.0 ± 0.3 ^c^	2.0 ± 0.3 ^c^	0.000	LOP
4	Pentanal	27.5 ± 4.0 ^e^	53.6 ± 6.9 ^d^	74.3 ± 1.3 ^ab^	80.7 ± 3.8 ^a^	60.4 ± 9.8 ^cd^	70.0 ± 5.6 ^bc^	65.7 ± 3.7 ^bc^	0.000	LOP
5	Hexanal	3.5 ± 1.0 ^e^	633.2 ± 13.3 ^a^	634.1 ± 14.7 ^a^	233.2 ± 8.9 ^c^	253.8 ± 10.1 ^b^	176.4 ± 10.5 ^d^	221.2 ± 15.4 ^c^	0.000	LOP
6	Heptanal	56.7 ± 3.4 ^c^	122.6 ± 15.7 ^b^	160.1 ± 2.1 ^a^	31.5 ± 1.7 ^d^	36.9 ± 1.2 ^d^	38.3 ± 3.6 ^d^	38.2 ± 7.2 ^d^	0.000	LOP
7	Octanal	84.7 ± 10.9 ^c^	182.0 ± 10.3 ^b^	230.9 ± 25.1 ^a^	62.4 ± 5.0 ^d^	86.4 ± 9.7 ^c^	50.5 ± 4.3 ^d^	54.9 ± 8.6 ^d^	0.000	LOP
8	(*Z*)-Hept-2-enal	N.D.	5.6 ± 0.2 ^b^	38.5 ± 9.0 ^a^	N.D.	N.D.	N.D.	N.D.	0.024	LOP
9	2-Methylpentanal	N.D.	3.6 ± 0.1 ^a^	N.D.	1.7 ± 0.3 ^b^	N.D.	N.D.	1.7 ± 0.01 ^b^	0.000	LOP
10	Nonanal	219.8 ± 13.3 ^cd^	1475.5 ± 124.6 ^a^	844.4 ± 94.8 ^b^	316.3 ± 5.8 ^c^	171.4 ± 17.8 ^d^	110.3 ± 7.9 ^d^	284.2 ± 11.9 ^c^	0.000	LOP
11	(*E*)-Oct-2-enal	24.6 ± 1.1 ^c^	76.0 ± 1.2 ^a^	42.2 ± 7.4 ^b^	3.2 ± 0.2 ^d^	5.5 ± 1.1 ^d^	6.6 ± 1.8 ^d^	8.3 ± 0.6 ^d^	0.000	LOP
12	Decanal	5.2 ± 0.6 ^bc^	9.1 ± 1.8 ^a^	8.0 ± 0.7 ^a^	4.4 ± 0.2 ^bc^	4.5 ± 0.6 ^bc^	4.2 ± 0.2 ^c^	5.9 ± 0.3 ^b^	0.001	LOP
13	Benzaldehyde	194.4 ± 17.0 ^c^	315.1 ± 13.5 ^b^	370.6 ± 52.7 ^a^	130.4 ± 12.0 ^de^	110.0 ± 8.5 ^e^	200.4 ± 23.3 ^c^	156.7 ± 11.5 ^cd^	0.000	AADP
14	(*E*)-Non-2-enal	7.5 ± 0.9 ^c^	11.3 ± 2.5 ^b^	15.1 ± 1.0 ^a^	1.6 ± 0.1 ^d^	1.8 ± 0.7 ^d^	2.4 ± 0.4 ^d^	1.9 ± 0.4 ^d^	0.000	LOP
15	(*Z*)-Dec-4-enal	11.9 ± 1.0 ^a^	14.1 ± 2.8 ^a^	14.2 ± 2.3 ^a^	3.4 ± 0.3 ^b^	3.6 ± 0.4 ^b^	4.0 ± 0.4 ^b^	3.0 ± 0.1 ^b^	0.000	LOP
16	Benzeneacetaldehyde	2.3 ± 0.1 ^d^	4.2 ± 0.9 ^c^	5.8 ± 1.6 ^be^	3.6 ± 0.2 ^cd^	7.4 ± 0.4 ^b^	9.5 ± 1.3 ^a^	6.5 ± 0.4 ^b^	0.000	MRP
17	(*E*)-Dec-2-enal	2.9 ± 0.2 ^b^	5.6 ± 1.1 ^a^	4.6 ± 1.0 ^a^	1.0 ± 0.1 ^c^	0.6 ± 0.02 ^c^	0.7 ± 0.1 ^c^	0.8 ± 0.1 ^c^	0.000	LOP
18	2-Butyloct-2-enal	N.D.	6.3 ± 0.3 ^b^	10.1 ± 1.4 ^a^	N.D.	N.D.	N.D.	N.D.	0.038	LOP
19	(*E,E*)-2,4-Nonadienal	2.7 ± 0.3 ^b^	6.0 ± 0.3 ^a^	5.8 ± 0.2 ^a^	N.D.	N.D.	N.D.	N.D.	0.000	LOP
20	Undec-2-enal	2.5 ± 0.2 ^a^	2.7 ± 0.7 ^a^	2.4 ± 0.3 ^a^	N.D.	N.D.	N.D.	N.D.	0.717	LOP
21	4-(1-Methylethyl)-benzaldehyde	N.D.	N.D.	N.D.	1.2 ± 0.1 ^a^	0.7 ± 0.2 ^c^	0.9 ± 0.1 ^b^	0.5 ± 0.1 ^c^	0.001	VFAB
22	(*E,E*)-2,4-Decadienal	7.3 ± 1.0 ^b^	10.0 ± 0.5 ^a^	7.9 ± 0.9 ^b^	0.8 ± 0.01 ^c^	N.D.	0.9 ± 0.2 ^c^	1.6 ± 0.1 ^c^	0.000	LOP
23	p-Anisaldehyde	N.D.	N.D.	N.D.	3.0 ± 0.2 ^b^	1.4 ± 0.2 ^d^	3.5 ± 0.2 ^a^	2.4 ± 0.4 ^c^	0.000	VFAB
24	Cinnamaldehyde	N.D.	N.D.	N.D.	2.0 ± 0.4 ^ab^	1.1 ± 0.2 ^b^	2.5 ± 0.2 ^a^	2.3 ± 0.8 ^a^	0.031	VFAB
25	Pentadecanal	33.9 ± 0.6 ^c^	127.9 ± 5.2 ^a^	66.2 ± 11.4 ^b^	32.4 ± 1.5 ^c^	18.4 ± 3.3 ^d^	79.8 ± 7.5 ^b^	28.7 ± 4.5 ^c^	0.000	LOP
26	Undec-10-enal	0.6 ± 0.1 ^b^	0.3 ± 0.1 ^c^	1.7 ± 0.2 ^a^	N.D.	N.D.	N.D.	N.D.	0.000	LOP
27	(*E*)-Octadec-9-enal	0.5 ± 0.01 ^d^	4.9 ± 0.5 ^a^	1.7 ± 0.1 ^b^	1.1 ± 0.1 ^c^	1.1 ± 0.1 ^c^	1.9 ± 0.1 ^b^	1.2 ± 0.4 ^c^	0.000	LOP
28	β-Cyclocitral	1.1 ± 0.02 ^b^	N.D.	N.D.	N.D.	4.2 ± 1.2 ^a^	3.5 ± 0.2 ^a^	1.9 ± 0.2 ^b^	0.001	LOP
29	Tetradecanal	N.D.	5.2 ± 0.8 ^a^	4.8 ± 0.5 ^a^	2.7 ± 0.1 ^b^	N.D.	N.D.	N.D.	0.003	LOP
30	Hexadecanal	N.D.	31.1 ± 0.8 ^c^	34.7 ± 0.2 ^b^	40.4 ± 0.8 ^a^	7.2 ± 0.3 ^f^	19.6 ± 1.1 ^d^	14.4 ± 0.6 ^e^	0.000	LOP
	**Alcohols (23)**	224.8 ± 14.1 ^f^	1073.6 ± 22.0 ^a^	525.9 ± 29.8 ^c^	756.6 ± 10.2 ^b^	347.5 ± 14.7 ^d^	358.1 ± 1.0 ^d^	310.2 ± 6.6 ^e^	0.000	
31	4-Methylpentan-1-ol	N.D.	N.D.	3.0 ± 0.2 ^b^	N.D.	N.D.	6.2 ± 0.2 ^a^	2.3 ± 0.1 ^c^	0.000	UO
32	Pent-1-en-3-ol	N.D.	17.1 ± 2.7 ^a^	16.2 ± 2.7 ^a^	N.D.	2.9 ± 0.4 ^c^	N.D.	N.D.	0.000	LOP
33	Butan-1-ol	1.5 ± 0.2 ^c^	N.D.	5.6 ± 1.1 ^a^	1.7 ± 0.1 ^bc^	1.8 ± 0.2 ^bc^	2.5 ± 0.5 ^bc^	2.5 ± 0.3 ^b^	0.000	LOP
34	1,8-Cineole	1.7 ± 0.8 ^e^	9.6 ± 0.8 ^d^	15.2 ± 1.8 ^d^	123.2 ± 11.5 ^a^	63.6 ± 1.4 ^b^	66.5 ± 0.4 ^b^	43.0 ± 1.1 ^c^	0.000	VFAB
35	Pentan-1-ol	32.2 ± 4.6 ^c^	268.2 ± 17.6 ^a^	165.8 ± 13.9 ^b^	26.6 ± 5.4 ^c^	26.3 ± 3.3 ^c^	29.2 ± 3.7 ^c^	28.6 ± 0.4 ^c^	0.000	LOP
36	(*Z*)-Pent-2-en-1-ol	N.D.	1.3 ± 0.3 ^a^	1.5 ± 0.1 ^a^	N.D.	N.D.	N.D.	N.D.	0.312	LOP
37	Hexan-1-ol	85.4 ± 3.5 ^b^	477.1 ± 18.3 ^a^	41.3 ± 2.5 ^c^	2.8 ± 0.6 ^e^	6.1 ± 0.7 ^e^	24.3 ± 2.6 ^d^	6.6 ± 0.5 ^e^	0.000	LOP
38	Oct-1-en-3-ol	1.5 ± 0.4 ^g^	130.0 ± 4.3 ^b^	137.0 ± 3.1 ^a^	50.6 ± 0.5 ^d^	59.4 ± 0.4 ^c^	27.7 ± 2.0 ^f^	40.2 ± 3.7 ^e^	0.000	LOP
39	Heptan-1-ol	10.3 ± 1.6 ^c^	31.6 ± 4.9 ^a^	24.9 ± 3.0 ^b^	3.3 ± 0.4 ^d^	4.8 ± 3.6 ^d^	2.5 ± 0.2 ^d^	3.7 ± 0.2 ^d^	0.000	LOP
40	2-Ethylhexan-1-ol	8.7 ± 2.3 ^b^	8.1 ± 1.3 ^b^	11.6 ± 0.9 ^a^	5.1 ± 0.2 ^c^	5.6 ± 0.3 ^c^	7.9 ± 0.3 ^b^	5.3 ± 0.8 ^c^	0.000	LOP
41	Linalool	N.D.	N.D.	N.D.	75.6 ± 5.9 ^a^	23.0 ± 2.0 ^b^	22.3 ± 0.9 ^b^	21.8 ± 2.7 ^b^	0.000	VFAB
42	Octan-1-ol	24.6 ± 0.4 ^c^	49.4 ± 5.8 ^a^	33.9 ± 3.3 ^b^	9.8 ± 0.4 ^d^	5.9 ± 0.7 ^d^	8.0 ± 0.9 ^d^	7.5 ± 0.2 ^d^	0.000	LOP
43	Butane-2,3-diol	N.D.	N.D.	4.0 ± 0.7 ^b^	N.D.	6.8 ± 0.7 ^a^	N.D.	N.D.	0.007	LOP
44	Terpinen-4-ol	N.D.	N.D.	N.D.	313.3 ± 5.9 ^a^	93.2 ± 6.8 ^b^	87.9 ± 4.0 ^b^	71.7 ± 3.5 ^c^	0.000	VFAB
45	(*E*)-Oct-2-en-1-ol	41.8 ± 6.2 ^c^	73.7 ± 11.9 ^a^	59.3 ± 10.0 ^b^	13.8 ± 1.4 ^d^	13.8 ± 1.7 ^d^	14.4 ± 2.0 ^d^	12.8 ± 0.4 ^d^	0.001	LOP
46	Nonan-1-ol	6.3 ± 0.8 ^a^	1.7 ± 0.2 ^b^	0.6 ± 0.01 ^c^	0.3 ± 0.1 ^c^	0.2 ± 0.01 ^c^	N.D.	0.2 ± 0.1 ^c^	0.000	LOP
47	Terpineol	N.D.	N.D.	N.D.	113.2 ± 3.5 ^a^	21.0 ± 2.1 ^d^	52.5 ± 1.9 ^b^	45.4 ± 1.4 ^c^	0.000	VFAB
48	(*E*)-Undec-2-en-1-ol	4.8 ± 0.8 ^ab^	5.5 ± 0.6 ^a^	5.2 ± 1.3 ^ab^	2.9 ± 0.2 ^cd^	3.8 ± 0.5 ^bc^	N.D.	2.2 ± 1.1 ^d^	0.002	LOP
49	(4R,6R)-cis-Carveol	N.D.	N.D.	N.D.	0.8 ± 0.02 ^a^	0.5 ± 0.1 ^bc^	0.6 ± 0.1 ^b^	0.4 ± 0.01 ^c^	0.001	VFAB
50	cis-Geraniol	N.D.	N.D.	N.D.	11.3 ± 1.7 ^a^	7.4 ± 0.6 ^b^	3.4 ± 0.1 ^c^	7.7 ± 0.8 ^b^	0.000	VFAB
51	Phenylethyl alcohol	2.7 ± 0.2 ^b^	N.D.	0.8 ± 0.02 ^d^	2.3 ± 0.1 ^b^	1.4 ± 0.3 ^c^	2.2 ± 0.1 ^b^	8.3 ± 0.5 ^a^	0.000	UO
52	Undecan-1-ol	3.0 ± 0.4	N.D.	N.D.	N.D.	N.D.	N.D.	N.D.	N.D.	VFRM
53	Dodecan-1-ol	0.3 ± 0.01 ^a^	0.3 ± 0.01 ^a^	N.D.	N.D.	N.D.	N.D.	N.D.	0.467	VFRM
	**Ketones (19)**	377.2 ± 17.6 ^de^	731.7 ± 42.2 ^b^	1411.9 ± 198.7 ^a^	530.8 ± 16.6 ^cd^	506.6 ± 7.2 ^cd^	323.6 ± 1.2 ^e^	555.9 ± 16.2 ^c^	0.000	
54	Butan-2-one	1.3 ± 0.3 ^d^	2.7 ± 0.9 ^d^	4.0 ± 0.7 ^cd^	30.5 ± 4.9 ^b^	35.0 ± 7.0 ^b^	9.3 ± 0.7 ^c^	49.0 ± 2.9 ^a^	0.000	VFAB
55	Butane-2,3-dione	12.5 ± 2.0 ^bc^	23.6 ± 1.7 ^a^	23.3 ± 3.0 ^a^	24.7 ± 2.1 ^a^	N.D.	15.0 ± 1.3 ^b^	11.1 ± 0.5 ^c^	0.000	MRP
56	Pentane-2,3-dione	N.D.	15.3 ± 2.9 ^a^	18.0 ± 2.6 ^a^	N.D.	N.D.	4.2 ± 0.6 ^c^	10.1 ± 0.4 ^b^	0.000	LOP
57	Heptan-2-one	25.2 ± 2.6 ^d^	60.3 ± 8.5 ^c^	118.5 ± 11.2 ^a^	19.4 ± 1.7 ^d^	N.D.	57.3 ± 4.1 ^c^	72.1 ± 5.8 ^b^	0.000	LOP
58	Octan-3-one	20.8 ± 1.0 ^a^	8.3 ± 2.5 ^c^	12.0 ± 1.1 ^b^	N.D.	2.1 ± 0.4 ^d^	6.0 ± 1.2 ^c^	N.D.	0.000	VFRM
59	Octan-2-one	7.2 ± 1.7 ^c^	12.0 ± 3.1 ^b^	18.3 ± 0.6 ^a^	N.D.	N.D.	N.D.	N.D.	0.002	LOP
60	1-Hydroxypropan-2-one	N.D.	20.7 ± 3.6 ^d^	57.7 ± 15.0 ^b^	29.7 ± 1.7 ^d^	54.7 ± 6.7 ^bc^	73.2 ± 2.2 ^a^	43.0 ± 2.2 ^c^	0.000	MRP
61	Octane-2,3-dione	303.7 ± 20.8 ^c^	578.6 ± 26.4 ^b^	1122.0 ± 184.4 ^a^	321.4 ± 9.1 ^c^	339.3 ± 7.4 ^c^	N.D.	297.9 ± 16.0 ^c^	0.000	LOP
62	6-Methylhept-5-en-2-one	N.D.	N.D.	N.D.	3.8 ± 0.5 ^c^	5.2 ± 1.0 ^a^	4.0 ± 0.6 ^bc^	2.9 ± 0.1 ^c^	0.019	VFAB
63	(*E*)-Oct-3-en-2-one	4.3 ± 0.6 ^c^	6.0 ± 0.5 ^b^	10.2 ± 0.6 ^a^	N.D.	N.D.	N.D.	N.D.	0.000	LOP
64	(*E,E*)-3,5-Octadien-2-one	N.D.	N.D.	24.1 ± 3.1	N.D.	N.D.	N.D.	N.D.	N.D.	LOP
65	6-Methyl-3,5-heptadiene-2-one	N.D.	N.D.	0.9 ± 0.2 ^a^	N.D.	N.D.	0.3 ± 0.01 ^b^	N.D.	0.003	LOP
66	Acetophenone	2.2 ± 0.2 ^bc^	4.2 ± 0.9 ^a^	2.9 ± 0.3 ^b^	2.6 ± 0.2 ^bc^	2.0 ± 0.1 ^c^	2.7 ± 0.5 ^bc^	2.3 ± 0.2 ^bc^	0.001	LOP
67	4-Isopropyl-2-cyclohexenone	N.D.	N.D.	N.D.	2.0 ± 0.1 ^a^	1.2 ± 0.1 ^b^	2.1 ± 0.3 ^a^	1.4 ± 0.2 ^b^	0.002	VFAB
68	Piperitone	N.D.	N.D.	N.D.	54.5 ± 2.9 ^a^	38.6 ± 4.2 ^b^	41.5 ± 3.3 ^b^	37.5 ± 1.8 ^b^	0.001	VFAB
69	Carvone	N.D.	N.D.	N.D.	2.7 ± 0.3 ^a^	1.6 ± 0.2 ^b^	N.D.	N.D.	0.005	VFAB
70	4-Phenylbutan-2-one	N.D.	N.D.	N.D.	1.9 ± 0.1 ^a^	N.D.	1.3 ± 0.1 ^c^	1.6 ± 0.1 ^b^	0.001	VFAB
71	4-Methoxyphenylacetone	N.D.	N.D.	N.D.	0.7 ± 0.02 ^a^	0.4 ± 0.1 ^b^	0.8 ± 0.1 ^a^	0.4 ± 0.0 ^b^	0.006	VFAB
72	Xanthoxylin	N.D.	N.D.	N.D.	36.9 ± 2.3 ^a^	26.5 ± 3.5 ^b^	39.8 ± 5.4 ^a^	26.6 ± 2.2 ^b^	0.003	VFAB
	**Hydrocarbons (25)**	280.1 ± 26.5 ^f^	2357.7 ±8 0.2 ^a^	1680.4 ± 41.0 ^e^	1946.6 ± 146.6 ^d^	2227.3 ± 43.0 ^b^	2087.4 ± 43.4 ^c^	2295.6 ± 30.1 ^ab^	0.000	
73	2-Methylbutane	N.D.	2.9 ± 0.6 ^a^	N.D.	1.6 ± 0.2 ^b^	N.D.	3.1 ± 0.2 ^a^	2.7 ± 0.3 ^a^	0.005	LOP
74	Benzene	N.D.	N.D.	5.4 ± 0.7 ^a^	2.8 ± 0.1 ^b^	N.D.	N.D.	N.D.	0.002	LOP
75	Toluene	10.3 ± 1.5 ^e^	179.8 ± 8.4 ^b^	238.8 ± 23.4 ^a^	119.9 ± 14.6 ^d^	141.4 ± 9.9 ^cd^	226.7 ± 15.0 ^a^	163.3 ± 8.8 ^bc^	0.000	AADP
76	Ethylbenzene	27.3 ± 0.8 ^b^	11.3 ± 0.4 ^d^	16.3 ± 1.5 ^c^	48.3 ± 5.9 ^a^	5.2 ± 1.8 ^e^	1.2 ± 0.2 ^e^	3.6 ± 0.06 ^e^	0.000	AADP
77	1,3-Dimethylbenzene	22.2 ± 2.0 ^d^	160.0 ± 7.1 ^b^	140.2 ± 30.3 ^b^	148.7 ± 15.1 ^b^	295.2 ± 9.6 ^a^	86.2 ± 13.8 ^c^	149.1 ± 10.6 ^b^	0.000	AADP
78	p-Xylene	N.D.	270.1 ± 4.8 ^d^	367.3 ± 10.0 ^c^	483.5 ± 80.8 ^b^	525.5 ± 21.4 ^b^	618.1 ± 10.2 ^a^	622.4 ± 18.3 ^a^	0.000	LOP
79	o-Xylene	168.3 ± 20.3 ^d^	992.9 ± 77.6 ^a^	221.5 ± 11.9 ^cd^	241.5 ± 19.1 ^c^	85.1 ± 3.5 ^e^	250.7 ± 17.4 ^c^	474.0 ± 17.9 ^b^	0.000	LOP
80	D-Limonene	N.D.	12.5 ± 1.8 ^c^	22.0 ± 0.7 ^b^	13.2 ± 1.7 ^c^	21.8 ± 2.4 ^b^	29.9 ± 3.1 ^a^	13.3 ± 0.7 ^c^	0.000	VFAB
81	Phellandrene	N.D.	N.D.	N.D.	3.4 ± 1.1 ^a^	2.3 ± 0.2 ^a^	N.D.	N.D.	0.218	VFAB
82	(Z)-3,7-Dimethyl-1,3,6-octatriene	N.D.	N.D.	N.D.	10.9 ± 0.8 ^b^	N.D.	13.4 ± 0.8 ^a^	N.D.	0.019	VFAB
83	1,3,5,7-Cyclooctatetraene	N.D.	456.8 ± 15.0 ^d^	435.3 ± 17.3 ^d^	445.6 ± 11.5 ^d^	621.5 ± 16.7 ^a^	567.1 ± 22.7 ^b^	530.5 ± 17.1 ^c^	0.000	LOP
84	Styrene	45.0 ± 6.3 ^d^	151.2 ± 17.9 ^b^	88.2 ± 11.2 ^cd^	323.8 ± 72.7 ^a^	328.1 ± 32.5 ^a^	113.3 ± 9.2 ^bc^	104.8 ±7.9 ^bc^	0.000	VFAB
85	o-Cymene	N.D.	N.D.	N.D.	27.3 ± 2.0 ^a^	20.6 ± 4.4 ^a^	N.D.	N.D.	0.101	VFAB
86	1,2,4-Trimethylbenzene	N.D.	10.5 ± 0.5 ^a^	10.9 ± 1.0 ^a^	8.4 ± 0.7 ^b^	N.D.	7.5 ± 1.4 ^b^	N.D.	0.007	LOP
87	m-Cymene	N.D.	N.D.	N.D.	0.4 ± 0.1 ^d^	77.2 ± 4.0 ^c^	93.6 ± 7.5 ^b^	128.9 ± 13.4 ^a^	0.000	VFAB
88	1,3,8-p-Menthatriene	N.D.	22.9 ± 2.2 ^a^	6.2 ± 0.5 ^c^	6.2 ± 1.0 ^c^	N.D.	9.2 ± 1.0 ^b^	7.9 ± 0.8 ^bc^	0.000	LOP
89	1-Methylindan	N.D.	N.D.	N.D.	7.5 ± 0.4 ^a^	7.7 ± 0.3 ^a^	6.9 ± 0.3 ^a^	7.5 ± 1.7 ^a^	0.123	VFAB
90	Naphthalene	3.5 ± 0.4 ^c^	N.D.	6.8 ± 0.4 ^b^	7.8 ± 1.1 ^ab^	7.2 ± 0.5 ^ab^	7.9 ± 0.4 ^ab^	8.2 ± 0.7 ^a^	0.000	UO
91	Decane	N.D.	6.4 ± 0.3 ^bc^	9.3 ± 0.2 ^a^	7.2 ± 0.7 ^bc^	7.5 ± 1.3 ^b^	5.9 ± 1.0 ^c^	6.3 ± 0.3 ^bc^	0.002	LOP
92	Dodecane	N.D.	26.3 ± 0.8 ^c^	34.9 ± 1.8 ^a^	31.2 ± 0.9 ^b^	34.2 ± 2.0 ^a^	19.6 ± 0.9 ^d^	29.6 ± 1.3 ^b^	0.000	LOP
93	Tridecane	N.D.	20.4 ± 1.6 ^a^	16.2 ± 2.1 ^b^	7.4 ± 1.2 ^d^	9.6 ± 0.2 ^c^	5.9 ± 0.1 ^d^	6.3 ± 0.1 ^d^	0.000	LOP
94	Tetradecane	N.D.	N.D.	21.4 ± 1.0 ^a^	N.D.	12.9 ± 1.3 ^b^	9.3 ± 0.4 ^c^	14.0 ± 0.6 ^b^	0.000	LOP
95	1,2,4,5-Tetramethylbenzene	3.5 ± 0.4 ^e^	24.4 ± 3.2 ^ab^	27.0 ± 1.6 ^a^	N.D.	21.7 ± 1.8 ^b^	8.4 ± 0.2 ^d^	17.4 ± 1.2 ^c^	0.000	UO
96	Pentadecane	N.D.	9.3 ± 0.3 ^b^	12.7 ± 0.9 ^a^	N.D.	N.D.	N.D.	N.D.	0.003	LOP
97	Longifolene	N.D.	N.D.	N.D.	N.D.	2.6 ± 0.05 ^b^	3.5 ± 1.4 ^b^	5.8 ± 0.6 ^a^	0.011	VFAB
	**Esters (13)**	1.9 ± 0.3 ^f^	222.8 ± 19.4 ^c^	440.8 ± 24.6 ^a^	120.8 ± 14.0 ^e^	166.2 ± 12.6 ^d^	399.9 ± 12.9 ^b^	205.8 ± 12.0 ^c^	0.000	
98	Ethyl acetate	N.D.	46.7 ± 3.9 ^c^	69.9 ± 3.8 ^a^	35.6 ± 7.3 ^d^	58.8 ± 1.2 ^b^	34.6 ± 4.2 ^d^	47.8 ± 1.0 ^c^	0.000	LOP
99	Ethenyl acetate	N.D.	60.9 ± 7.6 ^c^	210.3 ± 21.3 ^b^	26.8 ± 3.1 ^d^	2.4 ± 0.8 ^e^	247.0 ± 11.9 ^a^	27.0 ± 2.7 ^d^	0.000	LOP
100	Butyl butanoate	N.D.	8.2 ± 0.6 ^c^	12.3 ± 0.5 ^b^	3.3 ± 0.6 ^d^	N.D.	15.0 ± 1.2 ^a^	14.2 ± 1.2 ^a^	0.022	LOP
101	Isoamyl isobutyrate	N.D.	59.8 ± 8.3 ^c^	82.7 ± 5.3 ^a^	21.4 ± 2.9 ^d^	69.5 ± 9.4 ^bc^	69.2 ± 4.5 ^bc^	78.1 ± 7.4 ^ab^	0.000	LOP
102	Hexyl acetate	N.D.	N.D.	3.7 ± 0.3 ^b^	N.D.	2.5 ± 0.2 ^a^	N.D.	N.D.	0.004	UO
103	Hexyl butanoate	N.D.	2.4 ± 0.3 ^b^	3.7 ± 0.2 ^a^	0.9 ± 0.2 ^d^	1.5 ± 0.4 ^c^	1.4 ± 0.3 ^c^	3.2 ± 0.2 ^a^	0.000	LOP
104	1-(Acetyloxy)-2-propanone	N.D.	40.9 ± 2.7 ^b^	52.2 ± 7.0 ^a^	25.2 ± 1.4 ^c^	23.9 ± 2.3 ^c^	23.8 ± 1.9 ^c^	29.8 ± 3.2 ^c^	0.000	LOP
105	Butyrolactone	1.9 ± 0.3 ^d^	2.1 ± 0.5 ^bc^	3.9 ± 0.6 ^a^	1.7 ± 0.2 ^d^	2.6 ± 0.5 ^b^	2.1 ± 0.2 ^bc^	2.4 ± 0.1 ^b^	0.000	LOP
106	5-Ethyldihydro-2(3H)-furanone	N.D.	1.8 ± 0.4 ^b^	2.0 ± 0.3 ^a^	N.D.	0.5 ± 0.1 ^c^	0.6 ± 0.01 ^c^	0.5 ± 0.1 ^c^	0.000	LOP
107	Hexanolactone	N.D.	N.D.	0.1 ± 0.01 ^b^	0.8 ± 0.2 ^a^	N.D.	N.D.	0.9 ± 0.1 ^a^	0.000	LOP
108	Phenethyl acetate	N.D.	N.D.	N.D.	1.9 ± 0.1 ^a^	0.8 ± 0.2 ^b^	0.8 ± 0.2 ^b^	0.6 ± 0.1 ^b^	0.000	VFAB
109	Eugenol acetate	N.D.	N.D.	N.D.	3.0 ± 0.5 ^b^	3.7 ± 0.6 ^b^	5.1 ± 0.5 ^a^	1.3 ± 0.1 ^c^	0.000	VFAB
110	Coumarin	N.D.	N.D.	N.D.	0.2 ± 0.01 ^b^	N.D.	0.3 ± 0.01 ^a^	N.D.	0.041	VFAB
	**Ethers (5)**	2.8 ± 0.5 ^f^	16.5 ± 0.5 ^e^	16.9 ± 2.1 ^e^	116.8 ± 3.2 ^a^	37.1 ± 2.8 ^c^	41.7 ± 2.2 ^b^	25.0 ± 2.0 ^d^	0.000	
111	Anethole	2.8 ± 0.5 ^e^	16.5 ± 0.5 ^d^	16.6 ± 2.1 ^d^	80.7 ± 1.7 ^a^	33.3 ± 2.9 ^b^	35.1 ± 1.8 ^b^	21.8 ± 1.8 ^c^	0.000	VFAB
112	Estragole	N.D.	N.D.	0.3 ± 0.1 ^c^	31.5 ± 1.6 ^a^	1.5 ± 0.2 ^bc^	2.9 ± 0.3 ^b^	2.1 ± 0.5 ^b^	0.000	VFAB
113	Methyleugenol	N.D.	N.D.	N.D.	2.0 ± 0.1 ^a^	0.9 ± 0.2 ^c^	1.3 ± 0.1 ^b^	0.4 ± 0.01 ^d^	0.000	VFAB
114	Elemicin	N.D.	N.D.	N.D.	1.1 ± 0.2 ^a^	0.6 ± 0.1 ^b^	1.3 ± 0.1 ^a^	0.4 ± 0.01 ^b^	0.000	VFAB
115	Myristicin	N.D.	N.D.	N.D.	1.5 ± 0.2 ^a^	0.8 ± 0.1 ^c^	1.1 ± 0.04 ^b^	0.3 ± 0.02 ^d^	0.000	VFAB
	**Phenols (3)**	0.2 ± 0.01 ^e^	N.D.	N.D.	25.4 ± 2.3 ^b^	21.0 ± 0.6 ^c^	31.7 ± 4.5 ^a^	10.6 ± 0.3 ^d^	0.000	
116	Phenol	0.2 ± 0.01 ^a^	N.D.	N.D.	0.2 ± 0.02 ^a^	0.2 ± 0.02 ^a^	0.2 ± 0.01 ^a^	0.2 ± 0.02 ^a^	0.121	UO
117	Eugenol	N.D.	N.D.	N.D.	21.6 ± 2.8 ^b^	18.4 ± 0.7 ^b^	28.9 ± 4.5 ^a^	9.2 ± 0.5 ^c^	0.000	VFAB
118	trans-Isoeugenol	N.D.	N.D.	N.D.	3.6 ± 0.6 ^a^	2.4 ± 0.2 ^b^	2.6 ± 0.05 ^b^	1.8 ± 0.3 ^c^	0.000	VFAB
	**Acids (4)**	45.2 ± 3.2 ^a^	23.0 ± 1.3 ^bc^	45.0 ± 2.9 ^a^	19.9 ± 1.4 ^c^	13.6 ± 0.9 ^d^	24.9 ± 2.1 ^b^	24.2 ± 0.6 ^b^	0.000	
119	Acetic acid	N.D.	N.D.	15.6 ± 1.4 ^b^	14.7 ± 1.7 ^b^	6.9 ± 0.5 ^c^	18.6 ± 1.9 ^a^	16.6 ± 1.4 ^ab^	0.000	UO
120	Butanoic acid	10.5 ± 1.7	N.D.	N.D.	N.D.	N.D.	N.D.	N.D.	N.D.	VFRM
121	Pentanoic acid	32.1 ± 2.0 ^a^	N.D.	29.4 ± 2.0 ^a^	5.2 ± 0.3 ^c^	6.7 ± 0.7 ^b^	6.3 ± 0.4 ^b^	7.6 ± 1.2 ^b^	0.000	VFRM
122	Octanoic acid	2.6 ± 0.3	N.D.	N.D.	N.D.	N.D.	N.D.	N.D.	N.D.	VFRM
	**Heterocyclic and sulfur compounds (17)**	2.4 ± 0.2 ^c^	1192.7 ± 104.0 ^a^	1099.0 ± 198.8 ^a^	312.4 ± 32.1 ^b^	331.9 ± 17.2 ^b^	331.8 ± 2.3 ^b^	330.8 ± 19.7 ^b^	0.000	
123	2-Pentylfuran	N.D.	1071.5 ± 108.9 ^a^	975.5 ± 178.1 ^a^	249.0 ± 28.0 ^b^	250.1 ± 21.7 ^b^	234.3 ± 3.1 ^b^	234.2 ± 15.9 ^b^	0.001	LOP
124	3-(4-Methyl-3-pentenyl)-furan	N.D.	N.D.	N.D.	4.7 ± 1.0 ^b^	3.6 ± 0.03 ^c^	5.8 ± 0.1 ^a^	1.7 ± 0.1 ^d^	0.000	MRP
125	Furfural	N.D.	N.D.	1.9 ± 0.4 ^b^	2.1 ± 0.3 ^b^	2.9 ± 0.2 ^a^	3.0 ± 0.7 ^a^	3.4 ± 0.2 ^a^	0.005	MRP
126	2-Furanmethanol	N.D.	N.D.	N.D.	0.2 ± 0.1 ^c^	0.5 ± 0.1 ^b^	0.7 ± 0.02 ^a^	0.5 ± 0.1 ^b^	0.000	MRP
127	5-Hydroxymethylfurfural	N.D.	57.0 ± 6.8 ^a^	20.0 ± 0.7 ^b^	N.D.	N.D.	N.D.	N.D.	0.011	MRP
128	Safrole	N.D.	N.D.	N.D.	0.9 ± 0.2 ^a^	0.6 ± 0.1 ^b^	0.5 ± 0.03 ^b^	0.4 ± 0.1 ^b^	0.062	VFAB
129	Pyridine	0.9 ± 0.1 ^c^	1.5 ± 0.3 ^b^	0.8 ± 0.1 ^d^	1.4 ± 0.03 ^b^	0.8 ± 0.3 ^d^	1.1 ± 0.1 ^c^	2.7 ± 0.2 ^a^	0.000	MRP
130	2-Propylpyridine	N.D.	N.D.	2.9 ± 1.0	N.D.	N.D.	N.D.	N.D.	N.D.	MRP
131	2-Acetylpyrrole	N.D.	N.D.	N.D.	0.1 ± 0.01 ^c^	0.5 ± 0.2 ^b^	1.3 ± 0.1 ^a^	1.5 ± 0.3 ^a^	0.001	MRP
132	Methanethiol	1.0 ± 0.3 ^e^	47.5 ± 3.7 ^c^	84.1 ± 19.0 ^a^	32.9 ± 2.5 ^d^	51.1 ± 4.1 ^c^	68.5 ± 1.5 ^b^	56.4 ± 2.6 ^b^	0.000	AADP
133	Dimethyl disulfide	N.D.	0.9 ± 0.1 ^b^	5.5 ± 0.5 ^a^	N.D.	N.D.	N.D.	N.D.	0.000	AADP
134	3-Methylthiophene	N.D.	12.5 ± 1.0 ^c^	7.2 ± 1.3 ^d^	19.5 ± 2.7 ^b^	17.7 ± 1.0 ^b^	13.0 ± 1.5 ^c^	26.3 ± 1.0 ^a^	0.000	AADP
135	Dimethyl sulfone	0.5 ± 0.01 ^b^	N.D.	0.6 ± 0.02 ^a^	0.5 ± 0.1 ^b^	0.3 ± 0.02 ^c^	0.3 ± 0.01 ^c^	0.5 ± 0.1 ^b^	0.001	AADP
136	Dimethyl trisulfide	N.D.	1.4 ± 0.1 ^b^	N.D.	N.D.	1.5 ± 0.2 ^b^	2.1 ± 0.1 ^a^	1.6 ± 0.2 ^b^	0.001	MRP
137	2-Acetylthiazole	N.D.	N.D.	N.D.	0.9 ± 0.1 ^c^	2.0 ± 0.1 ^a^	0.9 ± 0.3 ^c^	1.4 ± 0.1 ^b^	0.000	MRP
138	2-Thiophenecarboxaldehyde	N.D.	N.D.	0.2 ± 0.02 ^a^	0.2 ± 0.01 ^a^	N.D.	N.D.	N.D.	0.786	MRP
139	Benzothiazole	N.D.	0.4 ± 0.1 ^a^	0.3 ± 0.01 ^b^	N.D.	0.3 ± 0.02 ^b^	0.3 ± 0.01 ^b^	0.2 ± 0.02 ^b^	0.067	MRP
	**Total**	1669.8 ± 97.1 ^c^	8096.5 ± 247.1 ^a^	8546.0 ± 623.7 ^a^	4788.4 ± 201.5 ^b^	4447.1 ± 87.1 ^b^	4414.2 ± 49.5 ^b^	4692.3 ± 58.2 ^b^	0.000	

Note: Results were expressed as mean value ± standard deviation. A row with different letters (a, b, c, d, e, f and g) is significantly different (*p* < 0.05). N.D. meant the volatile compounds were not found in the pork sample. FP, fresh pork; SP1, stewed pork with water; SP2: stewed pork with water and salt; SP3: stewed pork with water, salt and spices; SP4: stewed pork with water, salt, spices and soy sauce; SP5: stewed pork with water, salt, spices, soy sauce and sugar; SP6: stewed pork with water, salt, spices, soy sauce, sugar and cooking wine. Origin^1^: LOP, Lipid oxidation products; AADP, Amino acid degradation products; MRP, Maillard reaction products; VFAB, Volatiles from aged brine; VFRM, Volatiles from raw meat, UOAC, Unknown origin.

**Table 3:** Odour-active compounds (OAVs > 1) in the fresh and stewed pork.

No.	Compounds	^1^ Odour Descriptions; ^2^ Odour Thresholds (μg·kg^−1^)	FP	SP_1_	SP_2_	SP_3_	SP_4_	SP_5_	SP_6_	*p* Value
	**Aldehydes (14)**									
4	Pentanal	Almond, pungent; 9	3.1 ± 0.4 ^e^	6.0 ± 0.8 ^d^	8.3 ± 0.1 ^a b^	9.0 ± 0.4 ^a^	6.7 ± 1.1 ^cd^	7.8 ± 0.6 ^bc^	7.3 ± 0.4 ^bc^	0.000
5	Hexanal	Grass, fat; 4	0.9 ± 0.3 ^e^	158.3 ± 3.3 ^a^	158.5 ± 3.7 ^a^	58.3 ± 2.2 ^c^	63.5 ± 2.5 ^b^	44.1 ± 0.2 ^d^	55.3 ± 3.9 ^c^	0.000
6	Heptanal	Fat, citrus; 3	18.9 ± 1.1 ^c^	40.9 ± 5.3 ^b^	53.4 ± 0.7 ^a^	10.5 ± 0.6 ^d^	12.3 ± 0.4 ^d^	12.8 ± 1.2 ^d^	12.7 ± 2.4 ^d^	0.000
7	Octanal	Fat, lemon, green; 0.7	121.0 ± 15.5 ^c^	260.0 ± 14.8 ^b^	329.9 ± 35.8 ^a^	89.2 ± 7.1 ^d^	123.4 ± 13.8 ^c^	72.2 ± 6.1 ^d^	78.5 ± 12.3 ^d^	0.000
8	(*Z*)-Hept-2-enal	Fishy; 13.5	N.D.	0.4 ± 0.01 ^b^	2.9 ± 0.7 ^a^	N.D.	N.D.	N.D.	N.D.	0.024
10	Nonanal	Fat, citrus, green; 1	219.8 ± 13.3 ^cd^	844.4 ± 94.8 ^b^	1475.5 ± 124.6 ^a^	316.3 ± 5.8 ^c^	171.4 ± 17.8 ^d^	110.3 ± 7.9 ^d^	284.2 ± 11.9 ^c^	0.000
11	(*E*)-Oct-2-enal	Green, nut, fat; 3	8.2 ± 0.4 ^c^	25.3 ± 0.4 ^a^	14.1 ± 2.5 ^b^	1.1 ± 0.1 ^d^	1.8 ± 0.4 ^d^	2.2 ± 0.6 ^d^	2.8 ± 0.2 ^d^	0.000
12	Decanal	Soap, orange peel; 2	2.6 ± 0.3 ^bc^	4.5 ± 0.9 ^a^	4.0 ± 0.3 ^a^	2.2 ± 0.1 ^bc^	2.3 ± 0.3 ^bc^	2.1 ± 0.1 ^c^	3.0 ± 0.2 ^b^	0.001
14	(*E*)-Non-2-enal	Cucumber, green; 0.19	39.2 ± 5.0 ^c^	59.5 ± 13.3 ^b^	79.5 ± 5.2 ^a^	8.6 ± 0.6 ^d^	9.7 ± 3.6 ^d^	12.6 ± 1.9 ^d^	10.0 ± 2.4 ^d^	0.000
16	Benzeneacetaldehyde	Honey, sweet; 4	0.6 ± 0.03 ^d^	1.0 ± 0.2 ^c^	1.5 ± 0.4 ^b^	0.9 ± 0.1 ^cd^	1.8 ± 0.1 ^b^	2.4 ± 0.3 ^a^	1.6 ± 0.1 ^b^	0.000
17	(*E*)-Dec-2-enal	Orange; 0.3	9.8 ± 0.7 ^c^	18.6 ± 3.6 ^a^	15.4 ± 3.4 ^b^	3.3 ± 0.5 ^d^	2.0 ± 0.1 ^d^	2.5 ± 0.4 ^d^	2.7 ± 0.2 ^d^	0.000
19	(*E,E*)-2,4-Nonadienal	Geranium, pungent; 0.09	30.1 ± 3.0 ^b^	67.7 ± 3.2 ^a^	64.0 ± 2.5 ^a^	N.D.	N.D.	N.D.	N.D.	0.000
20	Undec-2-enal	Sweet; 0.78	3.3 ± 0.3 ^a^	3.5 ± 0.9 ^a^	3.1 ± 0.4 ^a^	N.D.	N.D.	N.D.	N.D.	0.717
22	(*E,E*)-2,4-Decadienal	Fried, wax, fat; 0.07	104.3 ± 14.7 ^b^	143.3 ± 7.6 ^a^	113.4 ± 13.1 ^b^	10.7 ± 0.6 ^c^	N.D.	12.4 ± 2.9 ^c^	22.7 ± 1.9 ^c^	0.000
	**Alcohols (4)**									
34	1,8-Cineole	Mint, sweet; 1	1.7 ± 0.8 ^e^	9.6 ± 0.8 ^d^	15.2 ± 1.8 ^d^	123.2 ± 11.5 ^a^	63.6 ± 1.4 ^b^	66.5 ± 0.4 ^b^	43.0 ± 1.1 ^c^	0.000
38	Oct-1-en-3-ol	Mushroom; 2	0.7 ± 0.2 ^g^	65.0 ± 2.2 ^b^	68.5 ± 1.5 ^a^	25.3 ± 0.3 ^d^	29.7 ± 0.2 ^c^	13.9 ± 1.0 ^f^	20.1 ± 1.8 ^e^	0.000
41	Linalool	Flower, lavender; 6	N.D.	N.D.	N.D.	12.6 ± 1.0 ^a^	3.8 ± 0.3 ^b^	3.7 ± 0.2 ^b^	3.6 ± 0.4 ^b^	0.000
45	(*E*)-Oct-2-en-1-ol	Soap, plastic; 50	0.8 ± 0.1 ^c^	1.5 ± 0.2 ^a^	1.2 ± 0.2 ^b^	0.3 ± 0.03 ^d^	0.3 ± 0.03 ^d^	0.3 ± 0.04 ^d^	0.3 ± 0.01 ^d^	0.000
	**Ketones (3)**									
55	Butane-2,3-dione	Butter; 4.37	2.9 ± 0.5 ^bc^	5.4 ± 0.4 ^a^	5.3 ± 0.7 ^a^	5.6 ± 0.5 ^a^	N.D.	3.4 ± 0.3 ^b^	2.5 ± 0.1 ^c^	0.000
60	1-Hydroxypropan-2-one	Sweet, pungent; 10	N.D.	2.1 ± 0.4 ^d^	5.8 ± 1.5 ^b^	3.0 ± 0.2 ^d^	5.5 ± 0.7 ^bc^	7.3 ± 0.2 ^a^	4.3 ± 0.2 ^c^	0.000
61	Octane-2,3-dione	Green, woody; 12	25.3 ± 1.7 ^c^	48.2 ± 2.2 ^b^	93.5 ± 15.4 ^a^	26.8 ± 0.8 ^c^	28.3 ± 0.6 ^c^	N.D.	24.8 ± 1.3 ^c^	0.000
	**Hydrocarbon (1)**									
80	D-Limonene	Citrus, mint; 10	N.D.	1.3 ± 0.2 ^c^	2.2 ± 0.1 ^b^	1.3 ± 0.2 ^c^	2.2 ± 0.2 ^b^	3.0 ± 0.3 ^a^	1.3 ± 0.1 ^c^	0.000
	**Ester (1)**									
98	Ethyl acetate	Pineapple; 5	N.D.	9.3 ± 0.8 ^c^	14.0 ± 0.8 ^a^	7.1 ± 1.5 ^d^	11.8 ± 0.2 ^b^	6.9 ± 0.8 ^d^	9.6 ± 0.2 ^c^	0.000
	**Ethers (2)**									
111	Anethole	Anissed-like; 15	0.2 ± 0.03 ^e^	1.1 ± 0.04 ^d^	1.1 ± 0.1 ^d^	5.4 ± 0.1 ^a^	2.2 ± 0.2 ^b^	2.3 ± 0.1 ^b^	1.5 ± 0.1 ^c^	0.000
112	Estragole	Licorice, anise; 6	N.D.	N.D.	0.1 ± 0.02 ^c^	5.2 ± 0.3 ^a^	0.3 ± 0.03 ^bc^	0.5 ± 0.1 ^b^	0.4 ± 0.1 ^b^	0.000
	**Phenol (1)**									
117	Eugenol	Clove, honey; 7.1	N.D.	N.D.	N.D.	3.0 ± 0.4 ^b^	2.6 ± 0.1 ^b^	4.1 ± 0.1 ^a^	1.3 ± 0.1 ^c^	0.000
	**Furan and sulfur compounds (3)**									
123	2-Pentylfuran	Green bean, butter; 6	N.D.	178.6 ± 18.1 ^a^	162.6 ± 29.7 ^a^	41.5 ± 4.7 ^b^	41.7 ± 3.6 ^b^	39.1 ± 0.5 ^b^	39.0 ± 2.4 ^b^	0.001
132	Methanethiol	Sulfur, gasoline, garlic; 1.05	0.9 ± 0.3 ^e^	45.2 ± 3.5 ^c^	80.1 ± 18.1 ^a^	31.3 ± 2.4 ^d^	48.7 ± 3.9 ^c^	65.3 ± 1.5 ^b^	53.7 ± 2.5 ^bc^	0.000
136	Dimethyl trisulfide	Sulfur, fish, cabbage; 0.01	N.D.	136.7 ± 9.1 ^b^	N.D.	N.D.	151.3 ± 15.9 ^b^	212.4 ± 12.5 ^a^	163.4 ± 17.2 ^b^	0.001
	**Total**		594.6 ± 58.6 ^d^	2136.6 ± 186.9 ^b^	2773.0 ± 263.3 ^a^	801.7 ± 42.0 ^c^	786.9 ± 67.4 ^c^	710.1 ± 43.1 ^cd^	849.6 ± 63.5 ^c^	0.000

Note: Each value is expressed as mean *±* SD; N.D. = not detected. ^a–e^ Different letters in the same row indicate that there is significant difference (*p <* 0.05, along the lines). FP, fresh pork; SP1, stewed pork with water; SP2: stewed pork with water and salt; SP3: stewed pork with water, salt and spices; SP4: stewed pork with water, salt, spices and soy sauce; SP5: stewed pork with water, salt, spices, soy sauce and sugar; SP6: stewed pork with water, salt, spices, soy sauce, sugar and cooking wine. ^1^ Odor descriptions were mainly gathered from online database, (http://www.flavornet.org, http://www.odour.org.uk). ^2^ Odor thresholds were mainly obtained from online database, (http://www.flavornet.org, http://www.odour.org.uk).

## Data Availability

The data presented in this study are available in the article and supplementary material.

## Supplementary Materials

The following are available online at https://www.mdpi.com/2304-8158/10/1/83/s1, Figure S1: Flow diagram of the stewed pork. Table S1: Information of the definitions and reference standards of odour attributes.

## References

[1] Yang Y., Pan D., Sun Y., Wang Y., Xu F., Cao J. (2019). ^1^H NMR-based metabolomics profiling and taste of stewed pork-hock in soy sauce. Food Res. Int..

[2] Han D., Mi S., Zhang C.H., Li J., Song H.L., Fauconnier M.L., Tyteca E. (2019). Characterization and discrimination of Chinese marinated pork hocks by volatile compound profiling using solid phase microextraction gas chromatography-mass spectrometry/olfactometry, electronic nose and chemometrics. Molecules.

[3] Zeng W., Wen W., Deng Y., Tian Y., Sun H., Sun Q. (2016). Chinese ethnic meat product: Continuity and development. Meat Sci..

[4] Qin Y.X., Cai D.D., Zhang D.N., Liu Y., Lai K.Q. (2019). Characteristics of volatile flavor component in stewed meat and meat broths prepared with repeatedly used broths containing star anise. J. Food Meas. Charact..

[5] Liu T.T., Yang T.S., Wu C.M. (2001). Changes of volatiles in soy sauce-stewed pork during cold storage and reheating. J. Sci. Food Agric..

[6] Overholt M.F., Mancini S., Galloway H.O., Preziuso G., Dilger A.C., Boler D.D. (2016). Effect of salt purity on lipid oxidation, sensory characteristics, and textural properties of fresh, ground pork patties. LWT Food Sci. Technol..

[7] Mariutti L.R., Bragagnolo N. (2017). Influence of salt on lipid oxidation in meat and seafood product: A review. Food Res. Int..

[8] Feng X., Ahn D.U. (2016). Volatile profile, lipid oxidation and protein oxidation of irradiated ready-to-eat cured turkey meat product. Radiat. Phys. Chem..

[9] Wang P., Hong Y., Ke W., Hu X., Chen F. (2017). Formation of heterocyclic amines in Chinese marinated meat: Effect of animal species and ingredient (rock candy, soy sauce and rice wine). J. Sci. Food Agric..

[10] Duan Y., Zheng F., Chen H., Huang M., Xie J., Chen F., Sun B. (2015). Analysis of volatiles in Dezhou Braised Chicken by comprehensive two-dimensional gas chromatography/high resolution-time of flight mass spectrometry. LWT Food Sci. Technol..

[11] Olivares A., Navarro J.L., Flores M. (2011). Effect of fat content on aroma generation during processing of dry fermented sausages. Meat Sci..

[12] Lu F., Kuhnle G.K., Cheng Q. (2018). The effect of common spices and meat type on the formation of heterocyclic amines and polycyclic aromatic hydrocarbons in deep-fried meatballs. Food Control..

[13] Yang H.S., Lee E.J., Moon S.H., Paik H.D., Ahn D.U. (2011). Addition of garlic or onion before irradiation on lipid oxidation, volatiles and sensory characteristics of cooked ground beef. Meat Sci..

[14] Sun L., Chen J., Li M., Liu Y., Zhao G. (2014). Effect of star anise (Illicium verum) on the volatile compounds of stewed chicken. J. Food Process. Eng..

[15] Zhao J., Wang M., Xie J., Zhao M., Hou L., Liang J.J., Wang S., Cheng J. (2017). Volatile flavor constituent in the pork broth of black-pig. Food Chem..

[16] Song S., Fan L., Xu X., Xu R., Jia Q., Feng T. (2019). Aroma Patterns Characterization of braised pork obtained from a novel ingredient by sensory-guided analysis and gas-chromatography-olfactometry. Foods.

[17] Huang X.H., Zheng X., Chen Z.H., Zhang Y.Y., Du M., Dong X.P., Qin D.L., Zhu B.W. (2019). Fresh and grilled eel volatile fingerprinting by E-nose, GC-O, GC-MS and GC × GC-QTOF combined with purge and trap and solvent-assisted flavor evaporation. Food Res. Int..

[18] Wang W., Feng X., Zhang D., Li B., Sun B., Tian H., Liu Y. (2018). Analysis of volatile compounds in Chinese dry-cured hams by comprehensive two-dimensional gas chromatography with high-resolution time-of-flight mass spectrometry. Meat Sci..

[19] Zhu Y., Lv H.P., Shao C.Y., Kang S., Zhang Y., Guo L., Dai W.D., Tan J.F., Peng Q.H., Lin Z. (2018). Identification of key odorant responsible for chestnut-like aroma quality of green teas. Food Res. Int..

[20] Li H., Li X., Zhang C.H., Wang J.Z., Tang C.H., Chen L.L. (2016). Flavor compounds and sensory profiles of a novel Chinese marinated chicken. J. Sci. Food Agric..

[21] Han D., Zhang C.H., Fauconnier M.L., Mi S. (2019). Characterization and differentiation of boiled pork from Tibetan, Sanmenxia and Duroc × (Landrace × Yorkshire) pigs by volatiles profiling and chemometrics analysis. Food Res. Int..

[22] Wang Y., Song H., Zhang Y., Tang J., Yu D. (2016). Determination of aroma compounds in pork broth produced by different processing methods. Flavour Fragr. J..

[23] Aaslyng M.D., Meinert L. (2017). Meat flavour in pork and beef-from animal to meal. Meat Sci..

[24] Yang Y., Sun Y., Pan D., Wang Y., Cao J. (2018). Effect of high pressure treatment on lipolysis-oxidation and volatiles of marinated pork meat in soy sauce. Meat Sci..

[25] Lorenzo J.M., Fonseca S. (2014). Volatile compounds of Celta dry-cured ‘lacon’ as affected by cross-breeding with Duroc and Landrace genotypes. J. Sci. Food Agric..

[26] Zou Y., Kang D., Liu R., Qi J., Zhou G., Zhang W. (2018). Effect of ultrasonic assisted cooking on the chemical profiles of taste and flavor of spiced beef. Ultrason. Sonochem..

[27] Petričević S., Radovčić N.M., Lukić K., Listeš E., Medić H. (2018). Differentiation of dry-cured hams from different processing methods by means of volatile compounds, physico-chemical and sensory analysis. Meat Sci..

[28] Jayasena D.D., Ahn D.U., Nam K.C., Jo C. (2013). Flavour chemistry of chicken meat: A review. Asian Australas. J. Anim. Sci..

[29] Gu S.Q., Wang X.C., Tao N.P., Wu N. (2013). Characterization of volatile compounds in different edible part of steamed Chinese mitten crab (Eriocheir sinensis). Food Res. Int..

[30] Gong H., Yang Z., Liu M., Shi Z., Li J., Chen W., Qiao X. (2017). Time-dependent categorization of volatile aroma compound formation in stewed Chinese spicy beef using electron nose profile coupled with thermal desorption GC–MS detection. Food Sci. Hum. Wellness.

[31] Toldrá F. (2017). Lawrie’s Meat Science.

[32] Van Ba H., Hwang I., Jeong D., Touseef A. (2012). Principle of meat aroma flavors and future prospect. Latest Res. Qual. Control..

[33] Pham A., Schilling M., Mikel W., Williams J., Martin J., Coggins P. (2008). Relationships between sensory descriptors, consumer acceptability and volatile flavor compounds of American dry-cured ham. Meat Sci..

[34] Lorenzo J.M., Montes R., Purriños L., Franco D. (2012). Effect of pork fat addition on the volatile compounds of foal dry-cured sausage. Meat Sci..

[35] Qi J., Liu D.Y., Zhou G.H., Xu X.L. (2017). Characteristic flavor of traditional soup made by stewing Chinese yellow-feather chickens. J. Food Sci..

[36] Ramírez R., Cava R. (2007). Volatile profiles of dry-cured meat product from three different Iberian × Duroc genotypes. J. Agric. Food Chem..

[37] Shahidi F. (1998). Flavor of Meat, Meat Product and Seafood.

[38] Lorenzo J.M., Franco D., Carballo J. (2014). Effect of the inclusion of chestnut in the finishing diet on volatile compounds during the manufacture of dry-cured “Lacón” from Celta pig breed. Meat Sci..

[39] Liu Y., Xu X.L., Ouyang G.F., Zhou G.H. (2006). Changes in volatile compounds of traditional Chinese Nanjing water-boiled salted duck during processing. J. Food Sci..

[40] Tai C.Y., Ho C.T. (1997). Influence of cysteine oxidation on thermal formation of Maillard aromas. J. Agric. Food Chem..

[41] Zhou X., Chong Y., Ding Y., Gu S., Liu L. (2016). Determination of the effect of different washing processes on aroma characteristics in silver carp mince by MMSE-GC-MS, e-nose and sensory evaluation. Food Chem..

[42] Lotfy S.N., Fadel H.H., El-Ghorab A.H., Shaheen M.S. (2015). Stability of encapsulated beef-like flavourings prepared from enzymatically hydrolysed mushroom proteins with other precursors under conventional and microwave heating. Food Chem..

