# Supplementary material for: Effect of Seasoning Addition on Volatile Composition and Sensory Properties of Stewed Pork
Source: Foods. 2021 Jan 4;10(1):83. doi: 10.3390/foods10010083 (PMC7824141; doi:10.3390/foods10010083)
Supplement: Supplementary file 1 [file foods-10-00083-s001.pdf]

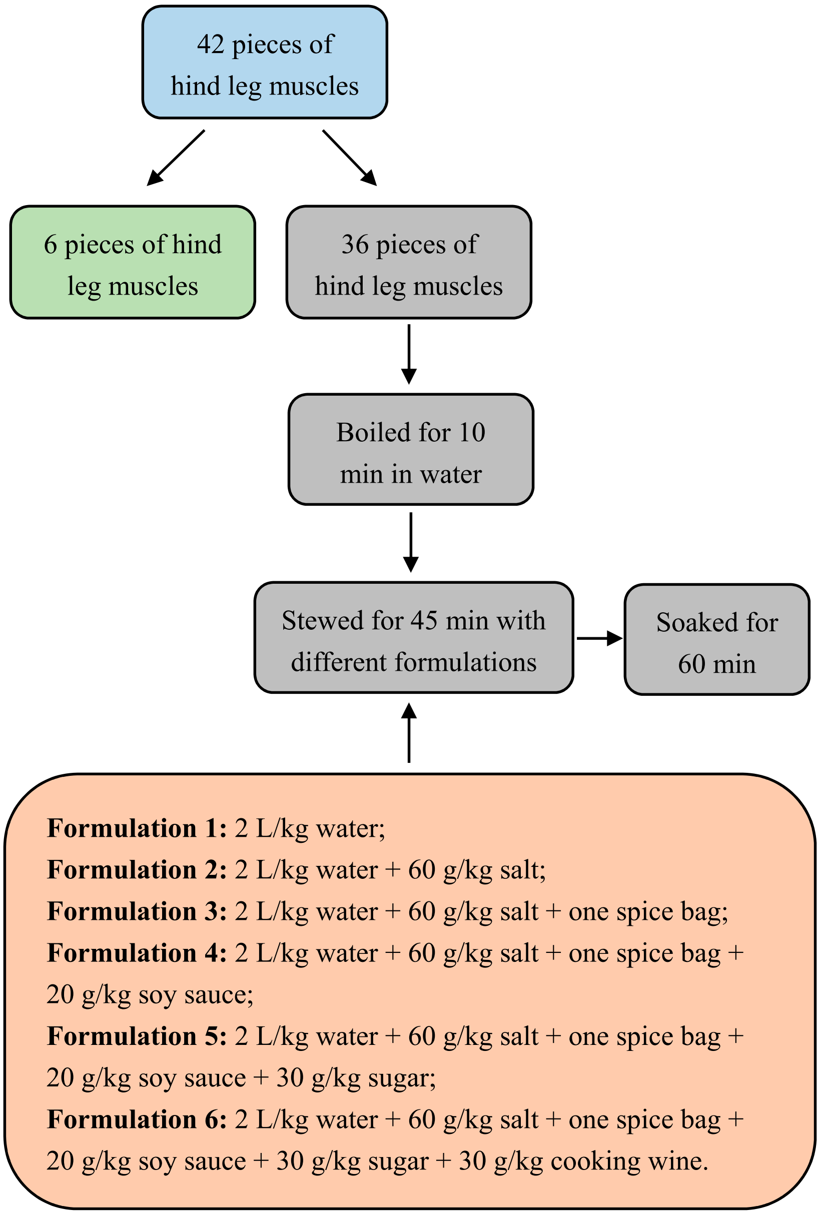

Fig. S1. Flow diagram of the stewed pork. The processing procedures consist of three steps including boiled for 10 min, stewed for 45 min and soaked for 60 min. The sampling points were chosen according to different stewing formulations.

Table S1. Information of the definitions and reference standards of odour attributes.

Odour attributes	Definitions	References (intensity)
Fatty	The smell associated with lard oil	Lard oil at 25°C (6.0)
Meaty	The smell associated with cooked pork	20.0 g of defatted pork in 60.0 mL of water was boiled for 1 h (8.0)
Caramel	The smell associated with burning white sugar	5.0 g of burning white sugar in 50.0 mL water (6.0)
Soy sauce	The smell associated with soy sauce	3.0 g of defatted pork in 50.0 mL of water (7.0)
Spicy	The smell associated with mixed spices	Freshly ground mixed spices (8.0)

Table S2. Identification of volatile compounds of the fresh and stewed pork by GC-MS/O and GC × GC-TOFMS.

No.	Compounds	GC-MS/O			GC × GC-TOFMS				Different processing methods							I
		RT (min)	RI[1]	RI[*2]	Peak Ⅰ (min)	Peak Ⅱ (s)	Similarity match/ Reverse match	Library probability	FP	SP_1	SP_2	SP_3	SP_4	SP_5	SP_6	method[3]
	**Aldehydes (30)**															
1	Acetaldehyde				5.2	1.64	945/972	21124	B[4]	N.D.[5]	N.D.	N.D.	N.D.	B	B	MS
2	Propanal				5.2	1.90	928/928	6177	N.D.	N.D.	B	N.D.	B	B	B	MS
3	Butanal				6.4	2.46	911/913	9365	B	B	B	B	B	B	B	MS
4	Pentanal	6.543	960	975	8.6	3.06	909/911	8986	B	A+B[6]	A+B	A+B	A+B	A+B	A+B	MS,RI,O
5	Hexanal	9.105	1065	1064	9.5	1.64	910/910	9236	A+B	A+B	A+B	A+B	A+B	A+B	A+B	MS,RI,O
6	Heptanal	11.928	1168	1182	16.0	3.34	910/910	8332	B	A+B	A+B	A+B	A+B	A+B	A+B	MS,RI,O
7	Octanal	14.881	1275	1287	20.5	3.50	937/944	9166	A+B	A+B	A+B	A+B	A+B	A+B	A+B	MS,RI,O
8	(*Z*)-Hept-2-enal				21.8	3.42	894/906	4130	N.D.	B	B	N.D.	N.D.	N.D.	N.D.	MS,O
9	2-Methylpentanal				24.8	4.28	866/892	6234	N.D.	B	N.D.	B	N.D.	N.D.	B	MS
10	Nonanal	17.671	1379	1388	25.0	3.28	918/918	7699	A+B	A+B	A+B	A+B	A+B	A+B	A+B	MS,RI,O
11	(*E*)-Oct-2-enal	18.574	1414	1425	26.4	3.22	917/918	5920	B	A+B	A+B	B	B	B	B	MS,RI,O
12	Decanal				29.4	3.86	897/898	6270	B	B	B	B	B	B	B	MS,O
13	Benzaldehyde	20.761	1502	1513	30.0	2.40	942/942	8474	A+B	A+B	A+B	A+B	A+B	A+B	A+B	MS,RI,O
14	(*E*)-Non-2-enal				30.8	3.16	921/921	6659	B	B	B	B	B	B	B	MS,O
15	(*Z*)-Dec-4-enal	21.280	1521	1544	31.1	3.42	882/885	6721	B	A+B	A+B	B	B	B	B	MS,RI,O
16	Benzeneacetaldehyde				34.8	2.42	921/937	7472	B	B	B	B	B	B	B	MS,O
17	(*E*)-Dec-2-enal				35.1	3.14	878/882	4859	B	B	B	B	B	B	B	MS,O
18	2-Butyloct-2-enal				36.2	3.78	856/887	7631	N.D.	B	B	N.D.	N.D.	N.D.	N.D.	MS
19	(*E,E*)-2,4-Nonadienal				37.2	2.80	849/878	4069	B	B	B	N.D.	N.D.	N.D.	N.D.	MS,O
20	Undec-2-enal				39.2	3.10	864/869	2780	B	B	B	N.D.	N.D.	N.D.	N.D.	MS
21	4-(1-Methylethyl)-benzaldehyde	26.715	1762	1759	40.0	2.78	869/872	7423	N.D.	N.D.	N.D.	A+B	A+B	A+B	A+B	MS,RI,O
22	(*E,E*)-2,4-Decadienal	27.377	1791	1790	41.2	2.72	877/910	5000	B	A+B	A+B	B	N.D.	B	B	MS,RI,O
23	p-Anisaldehyde	31.554	1999	1982	48.0	2.34	898/900	7404	N.D.	N.D.	N.D.	A+B	A+B	A+B	A+B	MS,RI,O

24	Cinnamaldehyde	31.834	2014	2040	48.5	2.32	856/903	3588	N.D.	N.D.	N.D.	A+B	A+B	A+B	A+B	MS,RI,O
25	Pentadecanal	31.817	2009	2024	52.3	3.08	918/929	3455	B	A+B	A+B	B	B	B	B	MS,RI
26	Undec-10-enal				59.3	3.04	868/874	3564	B	B	B	N.D.	N.D.	N.D.	N.D.	MS
27	(*E*)-Octadec-9-enal				59.2	3.00	858/868	1959	B	B	B	B	B	B	B	MS
28	β-Cyclocitral	23.242	1606	1598					A[7]	N.D.	N.D.	N.D.	A	A	A	MS,RI,O
29	Tetradecanal	29.739	1906	1927					N.D.	A	A	A	N.D.	N.D.	N.D.	MS,RI,O
30	Hexadecanal	33.813	2119	2137					N.D.	A	A	A	A	A	A	MS,RI
	**Alcohols (23)**															
31	4-Methylpentan-1-ol				4.2	1.78	830/992	6158	N.D.	N.D.	B	N.D.	N.D.	B	B	MS
32	Butan-1-ol				14.8	2.04	869/906	6144	B	N.D.	B	B	B	B	B	MS
33	Pent-1-en-3-ol				15.3	2.06	840/865	6976	N.D.	B	B	N.D.	B	N.D.	N.D.	MS
34	1,8-Cineole	12.540	1197	1209	16.9	4.48	837/837	7603	A+B	A+B	A+B	A+B	A+B	A+B	A+B	MS,RI,O
35	Pentan-1-ol	13.995	1242	1247	18.8	2.06	940/940	8241	B	A+B	A+B	B	B	B	B	MS,RI,O
36	(*Z*)-Pent-2-en-1-ol				21.4	2.12	847858	7080	N.D.	B	B	N.D.	N.D.	N.D.	N.D.	MS
37	Hexan-1-ol				23.0	2.22	906/906	5249	B	B	B	B	B	B	B	MS
38	Oct-1-en-3-ol	19.030	1432	1441	27.1	2.20	935/935	7624	A+B	A+B	A+B	A+B	A+B	A+B	A+B	MS,RI,O
39	Heptan-1-ol				27.3	2.30	914/914	5292	B	B	B	B	B	B	B	MS
40	2-Ethylhexan-1-ol				28.8	2.44	914/912	6606	B	B	B	B	B	B	B	MS
41	Linalool	21.400	1528	1545	31.2	2.42	908/908	7416	N.D.	N.D.	N.D.	A+B	A+B	A+B	A+B	MS,RI,O
42	Octan-1-ol	21.698	1541	1557	31.5	2.28	925/925	4288	A+B	A+B	A+B	A+B	A+B	A+B	A+B	MS,RI,O
43	Butane-2,3-diol				32.0	1.74	876/900	7628	N.D.	N.D.	B	N.D.	B	N.D.	N.D.	MS
44	Terpinen-4-ol	22.813	1588	1600	33.4	2.74	893/895	6364	N.D.	N.D.	N.D.	A+B	A+B	A+B	A+B	MS,RI,O
45	(*E*)-Oct-2-en-1-ol	23.014	1596	1605	33.7	2.16	896/896	6115	B	A+B	A+B	B	A+B	A+B	A+B	MS,RI,O
46	Nonan-1-ol				35.5	2.36	894/894	3409	B	B	B	B	B	N.D.	B	MS
47	Terpineol	24.924	1680	1688	36.9	2.54	926/929	7526	N.D.	N.D.	N.D.	A+B	A+B	A+B	A+B	MS,RI,O
48	(*E*)-Undec-2-en-1-ol				41.7	3.46	857/880	1377	B	B	B	B	B	N.D.	B	MS
49	(4R,6R)-cis-Carveol				41.8	2.30	859/863	7329	N.D.	N.D.	N.D.	B	B	B	B	MS
50	cis-Geraniol				42.2	2.2	867/867	9728	N.D.	N.D.	N.D.	B	B	B	B	MS
51	Phenylethyl alcohol	29.313	1886	1903	44.2	2.00	901/907	7082	B	N.D.	B	B	B	B	A+B	MS,RI,O

52	Undecan-1-ol				46.6	2.42	880/895	1095	B	N.D.	N.D.	N.D.	N.D.	N.D.	N.D.	MS
53	Dodecan-1-ol				53.2	2.42	844/851	1226	B	B	N.D.	N.D.	N.D.	N.D.	N.D.	MS
	**Ketones (19)**															
54	Butan-2-one				6.8	2.56	903/906	8450	B	B	B	B	B	B	B	MS
55	Butane-2,3-dione				8.5	2.06	954/977	7946	B	B	B	B	N.D.	B	B	MS,O
56	Pentane-2,3-dione				11.0	2.66	892/902	9222	N.D.	B	B	N.D.	N.D.	B	B	MS
57	Heptan-2-one				15.8	3.98	864/866	6987	B	B	B	B	N.D.	B	B	MS
58	Octan-3-one				19.0	4.70	864/871	6442	B	B	B	N.D.	B	B	N.D.	MS
59	Octan-2-one				20.3	1.82	825/836	3002	B	B	B	N.D.	N.D.	N.D.	N.D.	MS
60	1-Hydroxypropan-2-one				20.9	1.86	925/925	7017	N.D.	B	B	B	B	B	B	MS
61	Octane-2,3-dione				21.9	3.22	876/878	7035	B	B	B	B	B	N.D.	B	MS,O
62	6-Methylhept-5-en-2-one	16.095	1319	1336	22.5	3.40	850/850	9200	N.D.	N.D.	N.D.	A+B	A+B	A+B	A+B	MS,RI,O
63	Oct-3-en-2-one				25.5	3.30	871/889	8621	B	B	B	N.D.	N.D.	N.D.	N.D.	MS
64	(*E,E*)-3,5-Octadien-2-one				32.1	2.80	864/976	8185	N.D.	N.D.	B	N.D.	N.D.	N.D.	N.D.	MS
65	6-Methyl-3,5-heptadiene-2-one				32.9	2.92	826/849	4515	N.D.	N.D.	B	N.D.	N.D.	B	N.D.	MS
66	Acetophenone				35.0	2.58	886/898	4882	B	B	B	B	B	B	B	MS
67	4-Isopropyl-2-cyclohexenone				35.9	3.08	861/873	5963	N.D.	N.D.	N.D.	B	B	B	B	MS
68	Piperitone	25.663	1713	1739	38.1	2.96	894/906	8141	N.D.	N.D.	N.D.	A+B	A+B	A+B	A+B	MS,RI,O
69	Carvone				38.3	2.94	854/869	6159	N.D.	N.D.	N.D.	B	B	N.D.	N.D.	MS
70	4-Phenylbutan-2-one				42.6	2.58	849/857	8665	N.D.	N.D.	N.D.	B	N.D.	B	B	MS
71	4-Methoxyphenylacetone				52.2	2.34	803/825	7026	N.D.	N.D.	N.D.	B	B	B	B	MS
72	Xanthoxylin	40.619	2576		64.5	2.32	875/883	9405	N.D.	N.D.	N.D.	A+B	A+B	A+B	A+B	MS
	**Hydrocarbons (25)**															
73	2-Methylbutane				4.4	2.30	812/851	1512	N.D.	B	N.D.	B	N.D.	B	B	MS
74	Benzene				7.7	2.78	847/851	7126	N.D.	N.D.	B	B	N.D.	N.D.	N.D.	MS
75	Toluene	7.970	1022	1028	10.4	3.30	922/922	5019	A+B	A+B	A+B	A+B	A+B	A+B	A+B	MS,RI,O
76	Ethylbenzene				13.4	3.86	936/936	4866	B	B	B	B	B	B	B	MS
77	1,3-Dimethylbenzene				13.4	5.76	914/921	4126	B	B	B	B	B	B	B	MS
78	p-Xylene	10.735	1125	1130	13.9	3.62	943/948	2826	A	A+B	A+B	A+B	A+B	A+B	A+B	MS,RI,O

79	o-Xylene	11.953	1169	1183	14.0	3.56	947/947	3148	A+B	A+B	A+B	A+B	A+B	A+B	A+B	MS,RI,O
80	D-Limonene	12.411	1185	1192	16.3	5.48	899/900	7198	N.D.	A+B	A+B	A+B	A+B	A+B	A+B	MS,RI,O
81	Phellandrene				16.8	5.98	864/867	4478	N.D.	N.D.	N.D.	B	B	N.D.	N.D.	MS
82	(Z)-3,7-Dimethyl-1,3,6-octatriene				18.7	4.78	873/874	3365	N.D.	N.D.	N.D.	B	N.D.	B	N.D.	MS
83	1,3,5,7-Cyclooctatetraene				18.9	2.84	935/038	5329	N.D.	B	B	B	B	B	B	MS
84	Styrene	13.898	1239	1241	19.0	0.30	925/925	3546	A+B	A+B	A+B	A+B	A+B	A+B	A+B	MS,RI,O
85	o-Cymene	14.351	1255	1261	19.5	4.66	873/874	4480	N.D.	N.D.	A	A+B	A+B	A	A	MS,RI,O
86	1,2,4-Trimethylbenzene	14.625	1265	1278	20.0	4.28	858/868	2342	N.D.	B	B	A+B	N.D.	B	A	MS,RI
87	m-Cymene				26.7	3.86	897/914	2764	N.D.	N.D.	N.D.	B	B	B	B	MS
88	1,3,8-p-Menthatriene				26.7	3.92	851/869	1425	N.D.	B	B	B	N.D.	B	B	MS
89	1-Methylindan				29.2	4.02	839/842	4658	N.D.	N.D.	N.D.	B	B	B	B	MS
90	Naphthalene	25.784	1719	1740	38.4	2.90	904/924	6100	A+B	A	A+B	A+B	A+B	A+B	A+B	MS,RI,O
91	Decane	7.125	988	1000					N.D.	A	A	A	A	A	A	MS,RI
92	Dodecane	12.540	1190	1200					N.D.	A	A	A	A	A	A	MS,RI,O
93	Tridecane	15.291	1289	1293					N.D.	A	A	A	A	A	A	MS,RI
94	Tetradecane	17.930	1389	1400					N.D.	N.D.	A	N.D.	A	A	A	MS,RI
95	1,2,4,5-Tetramethylbenzene	18.712	1419	1435					A	A	A	N.D.	A	A	A	MS,RI
96	Pentadecane	20.461	1489	1500					N.D.	A	A	N.D.	N.D.	N.D.	N.D.	MS,RI
97	Longifolene	22.211	1562						N.D.	N.D.	N.D.	N.D.	A	A	A	MS
	**Esters (13)**															
98	Ethyl acetate				6.5	2.64	930/933	9285	N.D.	B	B	B	B	B	B	MS
99	Ethenyl acetate				6.6	2.32	930/935	6630	N.D.	B	B	B	B	B	B	MS
100	Butyl butanoate				17.4	5.06	870/871	4903	N.D.	B	B	B	N.D.	B	B	MS
101	Isoamyl isobutyrate				19.5	5.14	890/890	5765	N.D.	B	B	B	B	B	B	MS
102	Hexyl acetate				19.8	4.94	810/851	8386	N.D.	N.D.	B	N.D.	B	N.D.	N.D.	MS
103	Hexyl butanoate				26.0	4.94	850/878	7166	N.D.	B	B	B	B	B	B	MS
104	1-(Acetyloxy)-2-propanone				27.6	2.16	929/933	7829	N.D.	B	B	B	B	B	B	MS
105	Butyrolactone				34.0	2.20	925/937	9063	B	B	B	B	B	B	B	MS
106	5-Ethyldihydro-2(3H)-furanone				36.9	2.46	854/875	6660	N.D.	B	B	N.D.	B	B	B	MS

107	Hexanolactone				40.1	2.48	821/878	5374	N.D.	N.D.	B	B	N.D.	N.D.	B	MS
108	Phenethyl acetate				41.2	2.64	856/872	7724	N.D.	N.D.	N.D.	B	B	B	B	MS
109	Eugenol acetate				55.3	2.36	867/869	4599	N.D.	N.D.	N.D.	B	B	B	B	MS
110	Coumarin				60.4	2.30	807/865	8493	N.D.	N.D.	N.D.	B	N.D.	B	N.D.	MS
	**Ethers (5)**															
111	Anethole	27.631	1804	1809	41.6	2.70	911/917	5606	A+B	A+B	A+B	A+B	A+B	A+B	A+B	MS,RI,O
112	Estragole	24.232	1649	1655	41.7	2.74	872/887	5283	N.D.	A	B	B	A+B	A+B	A+B	MS,RI,O
113	Methyleugenol	31.297	1985	2006	47.8	2.58	863/868	9018	N.D.	N.D.	N.D.	A+B	A+B	A+B	A+B	MS,RI
114	Elemicin				54.5	2.48	834/838	8459	N.D.	N.D.	N.D.	B	B	B	B	MS
115	Myristicin	35.781	2235	2257	55.5	2.42	849/850	9720	N.D.	N.D.	N.D.	A+B	A+B	A+B	A+B	MS,RI,O
	**Phenols (3)**															
116	Phenol				47.2	1.70	907/907	9075	B	N.D.	N.D.	B	B	B	B	MS
117	Eugenol	34.130	2136	2141	52.5	2.04	909/910	4759	N.D.	N.D.	N.D.	A+B	A+B	A+B	A+B	MS,RI,O
118	trans-Isoeugenol	35.711	2227	2250					N.D.	N.D.	N.D.	A	A	A	A	MS,RI,O
	**Acids (4)**															
119	Acetic acid				22.4	1.74	936/951	9794	N.D.	N.D.	B	B	B	B	B	MS
120	Butanoic acid				29.3	1.86	840/876	9013	B	N.D.	N.D.	N.D.	N.D.	N.D.	N.D.	MS
121	Pentanoic acid				36.6	1.94	881/900	8026	B	N.D.	B	B	B	B	B	MS
122	Octanoic acid				44.0	1.96	856/856	7727	B	N.D.	N.D.	N.D.	N.D.	N.D.	N.D.	MS
	**Furan, N- or S-containing compounds (17)**															
123	Methanethiol				4.5	1.64	931/931	9863	B	B	B	B	B	B	B	MS,O
124	Dimethyl disulfide				11.5	4.76	867/878	9694	N.D.	B	B	N.D.	N.D.	N.D.	N.D.	MS
125	3-Methylthiophene				12.1	3.00	902/902	5186	N.D.	B	B	B	B	B	B	MS
126	Pyridine				16.1	3.14	848/874	8676	B	B	B	B	B	B	B	MS
127	2-Pentylfuran	13.276	1216	1231	17.8	3.94	880/880	6098	N.D.	A+B	A+B	A+B	A+B	A+B	A+B	MS,RI,O
128	Dimethyl trisulfide				24.2	3.40	839/852	9866	N.D.	B	N.D.	N.D.	B	B	B	MS,O
129	3-(4-Methyl-3-pentenyl)-furan				26.0	3.60	809/819	7961	N.D.	N.D.	N.D.	B	B	B	B	MS
130	Furfural				27.5	2.12	915/924	9442	N.D.	N.D.	B	B	B	B	B	MS
131	2-Propylpyridine				32.3	3.82	814/830	3931	N.D.	N.D.	B	N.D.	N.D.	N.D.	N.D.	MS

132	2-Acetylthiazole				34.8	2.38	860/882	9841	N.D.	N.D.	N.D.	B	B	B	B	MS
133	2-Furanmethanol				35.1	1.76	834/894	8184	N.D.	N.D.	N.D.	B	B	B	B	MS
134	2-Thiophenecarboxaldehyde				36.5	2.30	850/863	6376	N.D.	N.D.	B	B	N.D.	N.D.	N.D.	MS
135	Safrole				43.2	2.62	856/861	6791	N.D.	N.D.	N.D.	B	B	B	B	MS
136	Dimethyl sulfone				43.6	1.82	823/870	9776	B	N.D.	B	B	B	B	B	MS
137	Benzothiazole				45.7	2.54	826/836	9670	N.D.	B	B	N.D.	B	B	B	MS
138	2-Acetylpyrrole				46.1	1.88	831/863	9714	N.D.	N.D.	N.D.	B	B	B	B	MS
139	5-Hydroxymethylfurfural	38.915	2435	2512					N.D.	A	A	N.D.	N.D.	N.D.	N.D.	MS,RI,O

Note: FP, fresh pork; SP$_1$, stewed pork with water; SP$_2$: stewed pork with water and salt; SP$_3$: stewed pork with water, salt and spices; SP$_4$: stewed pork with water, salt, spices and soy sauce; SP$_5$: stewed pork with water, salt, spices, soy sauce and sugar; SP$_6$: stewed pork with water, salt, spices, soy sauce, sugar and cooking wine.

[1] RI: retention index.

[2] RI*: retention index from http://www.odour.org.uk/.

[3] Identification method: MS, mass spectrum comparison using NIST libraries; O, odor description.

[4] B: Volatile compounds were detected by GC × GC-TOFMS.

[5] N.D.: Volatile compounds were not detected by GC × GC-TOFMS or GC-MS/O.

[6] A+B: Volatile compounds were detected by both GC × GC-TOFMS and GC-MS/O.

[7] A: Volatile compounds were detected by GC-MS/O.
